# New Multicomponent Crystals of Antidiabetic Drug, Metformin: Mechanochemistry, Structural Studies, Biological Activity and Topological Analysis

**DOI:** 10.3390/ijms27073120

**Published:** 2026-03-30

**Authors:** Anita M. Grześkiewicz, Grzegorz Dutkiewicz, Paulina Pecyna, Marzena Gajecka, Maciej Kubicki

**Affiliations:** 1 Faculty of Chemistry, Adam Mickiewicz University, 61-614 Poznan, Poland; gdutkiew@amu.edu.pl; 2 Department of Genetics and Pharmaceutical Microbiology, Poznan University of Medical Sciences, 60-781 Poznan, Poland; pa.pecyna@ump.edu.pl (P.P.); gamar@man.poznan.pl (M.G.); 3 Institute of Human Genetics, Polish Academy of Sciences, 60-479 Poznan, Poland

**Keywords:** cocrystals, X-ray diffraction (XRD), metformin, mechanochemistry

## Abstract

Three multicomponent crystals of metformin were investigated to elucidate factors governing crystal architecture. Structures were determined by X-ray diffraction and analyzed using the Atoms-in-Molecules (AIM) approach, focusing on critical points and electron density topology. Three types of crystals were obtained: salt, cocrystal salt solvate and mixed salt with both organic and inorganic anions. Protonation of nitrogen atoms in metformin alters bond lengths and electron density, while strong intramolecular hydrogen bonds in hydrogenmaleate anions stabilize the structures and define the preferred anion geometry. Comparison with monoprotonated metformin revealed similar topological features despite differing protonation states. Mechanochemical synthesis via liquid-assisted grinding (LAG) enabled selective formation of specific crystalline forms, with the solvent type and acid polymorph influencing product distribution. These results highlight the critical roles of protonation, hydrogen bonding, and synthetic methodology in designing and controlling multicomponent metformin crystal structures.

## 1. Introduction

**Scheme 1:**
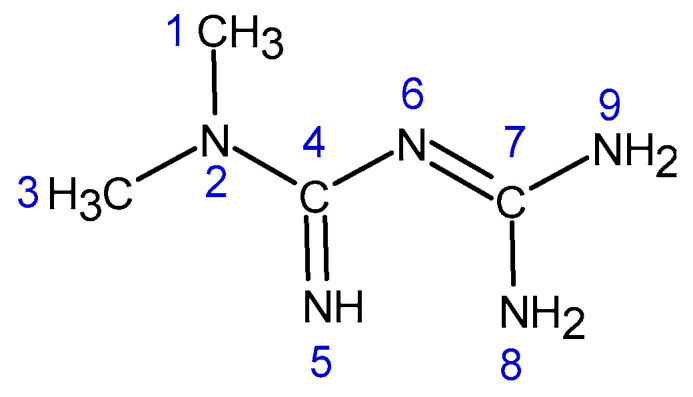
Structural formula of metformin.

Metformin (dimethylbiguanide (Scheme 1), hereinafter referred to as MF) is currently the first-line glucose-lowering drug for the treatment of type 2 diabetes. The medicinal properties of goat’s rue (*Galega officinalis*) have been known in traditional medicine for centuries; however, only in the 20th century—after a rather complex history of fluctuating interest, doubts and trials, cf., for instance, [1,2]—was metformin introduced into clinical use in Europe and the United States. At present, it ranks among the ten most widely prescribed drugs both in the United States and worldwide. Its extensive clinical use has stimulated intensive studies of its mechanisms of action (for instance, [3,4,5]).

The importance and widespread use of metformin have also sustained continuous interest in structural studies of its analogues, derivatives, salts, and cocrystals. As a consequence, an impressive number of metformin-containing compounds—currently, sixty-nine, while limited to the organic compounds—have been deposited in the Cambridge Structural Database (CSD [6]). Nearly all of these structures were reported after 2003, with more than 50% within the last five years, clearly demonstrating the intensified research activity in this area. A more detailed discussion regarding the database content will be presented later in this paper. It is also noteworthy that a substantial number of structures of metformin bound to proteins have been reported; according to the Protein Data Bank (PDB) [7], 71 such structures are currently available, with recent examples reported, for instance, in [8,9,10].

Therefore, studies of new multicomponent crystals containing metformin in different protonation states remain highly relevant, as do investigations of novel methods for their preparation, such as mechanochemical synthesis. Such studies also enable in-depth analyses of bonding in metformin and its salts using advanced theoretical approaches, for example, the Atoms-in-Molecules (AIM) method, to examine the topology of the electron density distribution.

Here, we present the results of our studies on three new multicomponent crystals (salt and salt solvates) of metformin with carboxylic acids, together with the details of their mechanochemical synthesis, topological analysis, and preliminary biological evaluation.

## 2. Results and Discussion

The three new salts of metformin with oxalic, malonic and mixed chloride–malonic acid were obtained and characterized in terms of X-ray diffraction, both powder and single-crystal.

### 2.1. CSD Analysis of MF Derivatives

**Figure 1:**
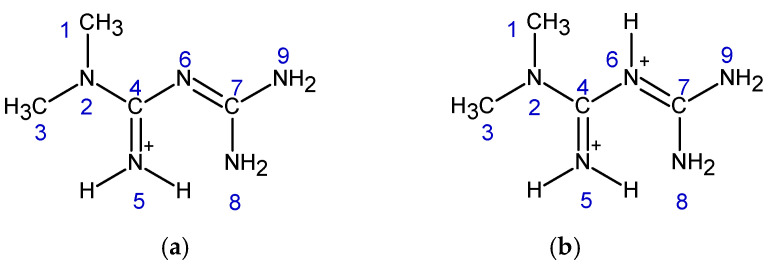
Mono- (**a**) and di- (**b**) cationic forms of metformin with the numbering scheme.

The structures of metformin-containing compounds deposited in the CSD can be divided into two groups: salts and solvated salts. Salts are considerably more common, accounting for nearly 70% of all reported structures. Within this group, two subgroups can be distinguished: mono- and diprotonated metformin (Figure 1), with an approximate ratio of 9:1.

This proportion differs in the second group (solvated salts), where monoprotonated forms still predominate; however, the ratio shifts to 0.55:0.45.

In monoprotonated salts, protonation occurs at the primary imine nitrogen atom (N5). In diprotonated salts, N5 is likewise protonated, while the second protonation takes place at the secondary imine nitrogen (N6). This assignment is further supported by an increase in the mean value of the valence angle C(4)–N(6)–C(7) upon protonation, from approximately 122° to approximately 128°.

For the studies reported here, salts with carboxylic acids are of primary importance. Among the five reported structures containing dicarboxylic acids (oxalic [11] and 1,4-phenylenedioxalamic [12]), metformin is doubly protonated in two cases, whereas in the remaining three—namely, the salts with malonic [13], succinic [14], and maleic acid [13]—it is monoprotonated.

In these salts, metformin can adopt two distinct conformations, defined by the value of the N(2)–C(4)–N(6)–C(7) torsion angle (Figure S1, Supplementary Materials). In type I, the absolute value of this angle falls within the range of 30–90°, with a maximum of around 55°, whereas in type II, the torsion angle ranges from 122° to 163°, with a maximum of around 147°. The latter conformation is clearly preferred. Indeed, in all salts with dicarboxylic acids reported to date, metformin adopts the type II conformation.

### 2.2. Structural Studies 

#### 2.2.1. MF^2+^·2OX^−^·H_2_O·0.5OX (**1**)

The structure of the multicomponent crystal (MCC) composed of doubly protonated metformin, doubly deprotonated oxalate dianion, and water in a 1:1:1 molar ratio was reported in 2004 [11]. According to the classification proposed by Grothe et al. [15], this structure can be unambiguously described as a salt solvate, MF^2+^·OX^2−^·H_2_O.

**Figure 2:**
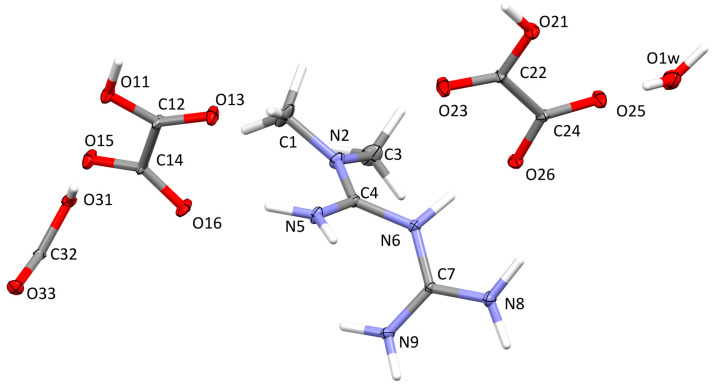
Perspective view of the asymmetric part of **1** with the atom-labeling scheme. Displacement ellipsoids are drawn at the 50% probability level; hydrogen atoms are represented as capped sticks.

In the present study, we obtained another MCC apparently composed of the same components, but in a different molar ratio. However, detailed structural analysis reveals that the fundamental building units differ from those previously reported. The asymmetric unit contains one metforminium dication, two singly deprotonated hydrogenoxalate anions, one half of a neutral oxalic acid molecule, and one water molecule (Figure 2). Accordingly, the overall formula may be written as 2MF^2+^·4OX^−^·OX·2H_2_O. Using the same classification scheme, this compound should be regarded as a cocrystal salt solvate. A search of the Cambridge Structural Database (CSD) indicates that this structure is the only reported metformin-containing compound that can be assigned to this category.

In compound **1**, the N(3)–C(4)–N(6)–C(7) torsion angle is 143.0(1)°, indicating that the metformin moiety adopts a type II conformation (vide supra). The terminal C–N bond lengths fall within the range of 1.3174(13)–1.3198(14) Å, consistent with significant electron delocalization. The overall bond length pattern suggests delocalization over most of the metformin framework, with the exception of the C1–N2 and C3–N2 bonds. A more detailed discussion is provided in the subsequent sections, where the topology of the electron density distribution is analyzed.

**Figure 3:**
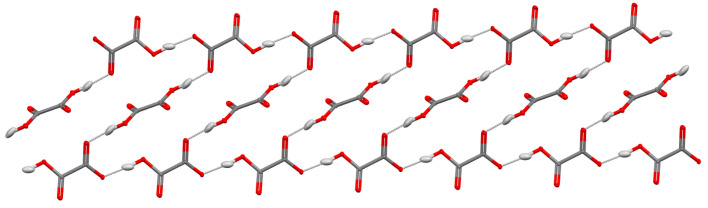
Hydrogen-bonding pattern between hydrogenoxalate anions and oxalic acid in compound **1**.

Owing to double protonation, the metforminium cation acts exclusively as a hydrogen-bond donor. In contrast, the hydrogenoxalate anions, neutral oxalic acid molecules, and water molecules may function both as donors and acceptors within the hydrogen-bonding network. The hydrogenoxalate anions form C(5) hydrogen-bonded chains extending along the [100] direction. These chains are interconnected by neutral oxalic acid molecules, resulting in the formation of large R_6_^6^(28) ring motifs (Figure 3). Hydrogen bond data are listed in Table 1.

Given the abundance of efficient hydrogen-bond donors and acceptors, the formation of diverse hydrogen-bonding motifs is highly favored. Consequently, packing analysis reveals that the multiplicity of donor and acceptor sites gives rise to a highly complex and extended hydrogen-bonding network.

#### 2.2.2. Metformin with Maleic Acid

The structure of the simple 1:1 salt of metformin with double deprotonated maleic acid [13] is deposited with the CSD [6]. We obtained two new salts of MF and the hydrogenmaleate anion, described in the following subchapters.

##### MF^2+^·2MAL^−^ (**2**)

**Figure 4:**
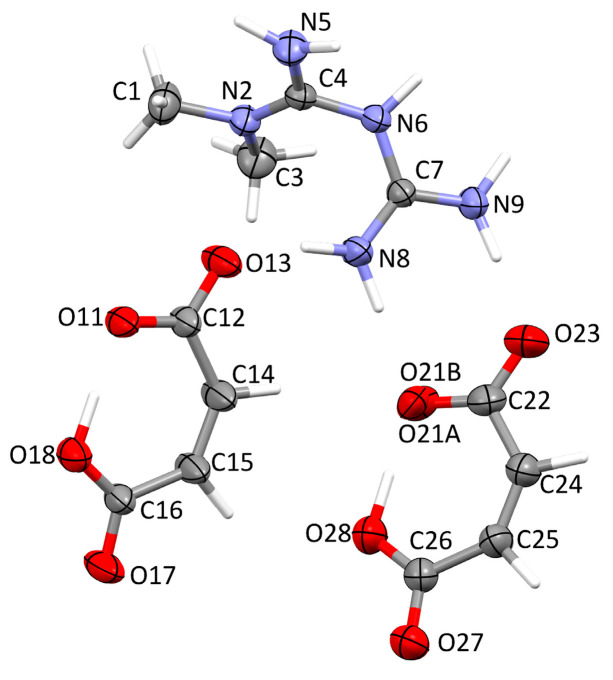
Asymmetric unit of compound **2** with the atom-labeling scheme. Displacement ellipsoids are drawn at the 50% probability level; hydrogen atoms are represented as capped sticks.

Structure **2** represents a simple salt composed of a metforminium dication and two hydrogenmaleate monoanions, corresponding to a 1:2 stoichiometric ratio (Figure 4). Double protonation suppresses the hydrogen-bond acceptor capability of metformin, which leads to a pronounced modification of the crystal packing. The structure consists of alternating layers of protonated metformin and hydrogenmaleate anions. The packing arrangement is governed predominantly by N–H···O hydrogen bonds, in which the metforminium cation acts exclusively as the donor and the oxygen atoms of the anions serve as acceptors.

**Figure 5:**
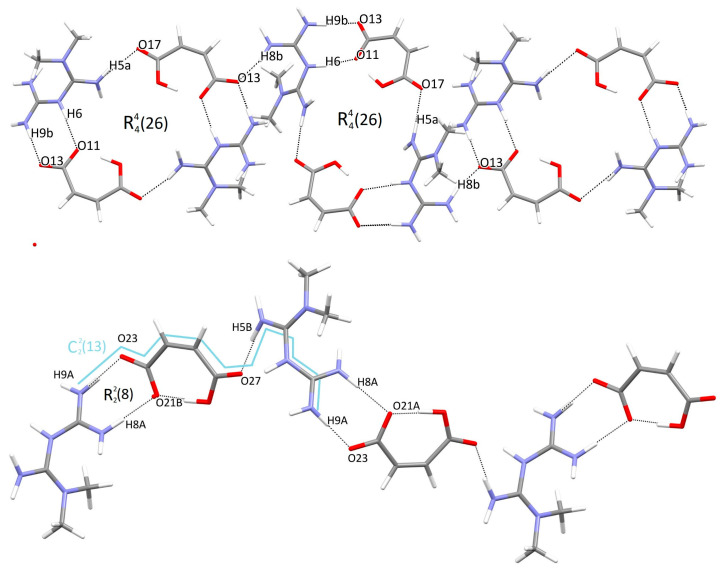
Selected hydrogen bond pattern in **2** (cf. text).

The metforminium cation forms hydrogen bonds with both symmetry-independent hydrogenmaleate anions. In the case of the first anion, the primary supramolecular motif consists of centrosymmetric R_4_^4^(26) and R_4_^4^(22) ring patterns (excluding the intramolecular hydrogen bond; Figure 5, top). These motifs are further linked by N8–H···O13 hydrogen bonds to generate a one-dimensional chain.

In contrast, interaction with the second hydrogenmaleate anion results in the formation of a C_2_^2^(13)[R_2_^2^(8)] chain motif via N8/N9–H···O21/O23 and N5–H···O27 hydrogen bonds (Figure 5, bottom). The corresponding geometric parameters are listed in Table 2. In addition to these strong N–H···O interactions, the metforminium cation participates in C–H···O hydrogen bonds with both symmetry-independent hydrogenmaleate anions. The structure is further stabilized by weaker intermolecular contacts, including O···π, π···π, O···O, and C–H···N interactions.

Compound **2** constitutes the first reported example of a metformin salt with a carboxylic acid in which the metformin moiety adopts a type I conformation, characterized by an NCNC torsion angle of −55.13°. Both hydrogenmaleate anions exhibit very strong intramolecular hydrogen bonds (E_int_ = −147.2 and −163.3 kJ·mol^−1^; interaction energies calculated according to [16]). These interactions are significantly stronger than the next most stabilizing intermolecular contact in the structure, namely, the N9–H···O23 hydrogen bond (E_int_ = −42.8 kJ·mol^−1^). A more detailed discussion of this interaction is provided in Section 2.3. 

##### MF^2+^·MAL^−^·Cl^−^·H_2_O (**3**)

**Figure 6:**
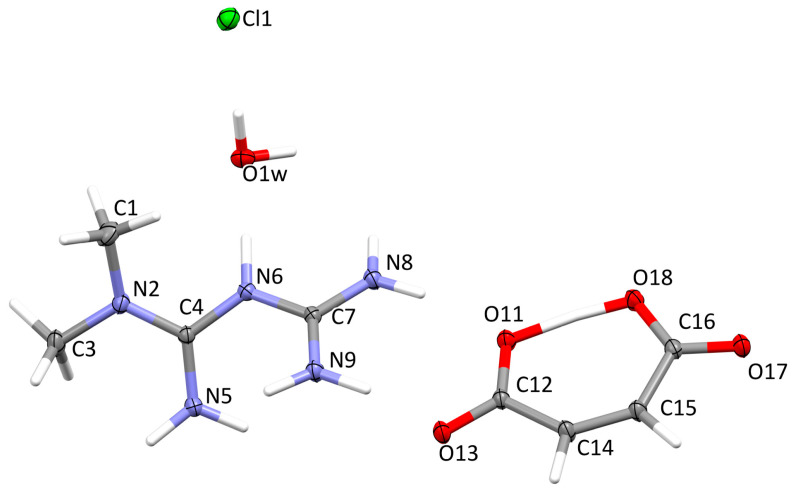
Asymmetric unit of compound **3** with atom-labeling scheme. Displacement ellipsoids are drawn at the 50% probability level; hydrogen atoms are represented as capped sticks.

Compound **3** can be classified as a mixed salt solvate, comprising deprotonated metformin, two distinct counteranions—hydrogenmaleate and chloride—and one water molecule (Figure 6). A search of the Cambridge Structural Database (CSD) reveals only a single previously reported example of a mixed salt containing two different anions in the crystal lattice (nitrate–perchlorate) [17]. Therefore, the present structure represents the first example of a metformin salt containing two different anions, one organic and one inorganic.

The structure contains both efficient hydrogen-bond donors and acceptors, and hydrogen bonding is the primary determinant of the crystal packing (Table 3). As observed in compound **2**, the strongest interaction is an intramolecular hydrogen bond within the hydrogenmaleate anion, with an interaction energy of E_int_ = −193.28 kJ·mol^−1^, exceeding that found in compound **2**. The second strongest interaction, N6–H···O1w, exhibits E_in_t = −71.6 kJ·mol^−1^, which is more than 2.5 times weaker than the primary intramolecular hydrogen bond. Geometric parameters for compounds **1**–**3** has been summarized in Tables S1–S3 (Supplementary Materials).

### 2.3. Topological Analysis

To analyze in detail the factors governing crystal architecture—a critical aspect in the formation of multicomponent crystals—the Atoms-in-Molecules (AIM) approach was applied to examine the electron density distribution [18]. Specifically, critical points (CPs) were calculated for all structures, analyzed, and compared with the fully experimental electron density distribution reported for metformin hydrochloride based on high-resolution X-ray diffraction data [19].

The models presented here were initially refined using Hirshfeld Atom Refinement (HAR) [20] and, after successful convergence, further transformed into the multipolar Hansen–Coppens model [21] by transferring multipolar parameters from the ELMAM2 database [22]. A comparison was attempted between (a) diprotonated metformin structures (**1**–**3**) obtained using this procedure and (b) purely experimentally derived data available for the monoprotonated cation [19]. The two approaches reveal largely similar features.

**Figure 7:**
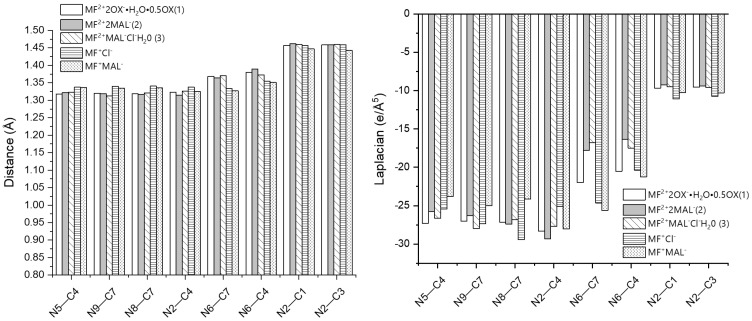
Selected characteristics of bonds in metformin cation.

The largest differences arise from the protonation states, particularly in the N6–C7 and N6–C4 bonds (cf. Figure 7). Protonation of N6 in compounds **1**–**3** leads to a slight elongation of the delocalized bonds involving these atoms, resulting in a significant decrease in electron density at the corresponding CPs compared with metformin hydrochloride. Notably, the Laplacian value at the N6–C4 bond CP in compound **1** is closer to that of the unprotonated form than the corresponding values in compounds **2** and **3**. This observation correlates with the relatively low overall energy of the hydrogen bonds donated by N6–H6 in **1** (E_int_ = −202.4 kJ·mol^−1^) compared with compounds **2** (E_int_ = −249.2 kJ·mol^−1^) and **3** (E_int_ = −228.5 kJ·mol^−1^).

**Figure 8:**
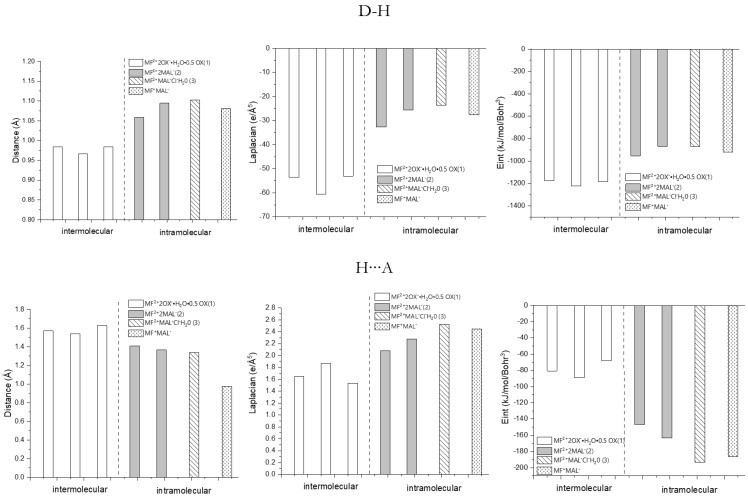
Selected characteristics of intra- and intermolecular hydrogen bonds in metformin salts. The dashed line separates intramolecular interactions from intermolecular interactions.

In all structures containing maleic acid (**2**, **3**, and MF^+^·MAL^−^ [19]), the counterions are present as hydrogenmaleate, even for doubly protonated metformin, where two hydrogenmaleate anions compensate the charge, rather than a single doubly deprotonated maleate. In each case, protonation of the oxygen atom in the hydrogenmaleate anion is associated with a very strong intramolecular hydrogen bond (Figure 8).

Given the pronounced preference of maleic acid to form hydrogenmaleate anions in metformin salts, we examined the intramolecular hydrogen bond in detail. The donor–acceptor distances are similar (2.426–2.465 Å), typical for the hydrogenmaleate anion. Analysis of bond critical points (BCPs) for the intramolecular hydrogen bond (Figure 8) shows that the covalent H–O bond is significantly elongated, resulting in weaker bonds compared with standard O–H bonds. Conversely, the H···acceptor distance is short, with characteristic BCP properties. Comparison of the intramolecular hydrogen bonds in compounds **1**, **2**, and MF^+^·MAL^−^ with the intermolecular hydrogen bond in compound **1** shows that in hydrogenmaleate, the D–H bond elongates as the H···A distance decreases relative to the oxalic acid system. Other parameters indicative of bond strength, such as electron density and Laplacian at the CP, are substantially larger for the intramolecular hydrogen bond than for the intermolecular one. Calculated interaction energies [16] indicate that the intramolecular O–H···O hydrogen bond in hydrogenmaleate is approximately twice as strong as the intermolecular hydrogen bond in compound **1**. Although the intermolecular hydrogen bond in compound **1** meets the criteria for a strong hydrogen bond according to Munshi and Guru Row [23], these results suggest that the unusually strong intramolecular hydrogen bond may favor the formation of hydrogenmaleate over the fully deprotonated maleate anion, likely due to the optimal anion geometry. The characteristics of bond critical points found for compounds **1**–**3** has been enclose in Supplementary Materials (Tables S4–S6).

### 2.4. Mechanochemistry

Crystals of compounds **1**–**3** were obtained both by solution evaporation and by liquid-assisted mechanochemistry (LAG), a method increasingly employed in multicomponent drug design due to its simplicity, reproducibility, and minimal solvent usage. Powder X-ray diffractograms of all multicomponent crystals are provided in the Supplementary Materials. The full synthetic procedures are described in the Synthesis Section 3.1.1 and Section 3.1.2.

Compound **1** was obtained as a pure phase, whereas compounds **2** and **3** were typically obtained as mixtures. LAG reactions with an appropriate solvent allowed selective direction of the reaction toward the desired product; however, minor impurities of the undesired forms were still present. Complete isolation of pure phases using LAG was not achieved.

For compound **1**, the choice of solvent appeared less critical than the substrates. Reactions conducted with either dimethyl sulfoxide or water as liquid assistance yielded similar results (Figure S2) when hydrated oxalic acid was used. Changing the form of oxalic acid affected the outcome: using pure β-polymorph oxalic acid did not yield compound **1**, whereas using a mixture of α-polymorph and its hydrate produced compound **1** along with metforminium chloride and oxalic acid hydrates as side products (Figures S2 and S3). After purification, the diffractograms indicated the presence of both crystalline and amorphous phases (Figure S4).

For multicomponent crystals containing maleic acid, LAG was particularly critical, as crystallizations from solution produced both compounds **2** and **3** regardless of the solvent (Figure S5). Under LAG, solvent choice influenced the product distribution: ethanol predominantly yielded compound **2** (Figure S6), while water, serving both as solvent and reactant, favored the formation of compound **3** (Figure S7).

### 2.5. Biological Studies

Compound **1**, obtained from the mechanochemical reaction, metformin and oxalic acid (α-polymorph) mixed with hydrate of oxalic acid, which was used for reaction without prior purification, were tested for antibacterial activity against the reference and clinical Gram-positive bacteria: *Staphylococcus aureus* ATCC 29213 and *Staphylococcus aureus* MRSA (methicillin-resistant *Staphylococcus aureus*—clinical); *Enterococcus faecium* ATCC 700 221—VRE (vancomycin-resistant Enterococcus); *Enterococcus faecalis* ATCC 19433; Gram-negative strains *Escherichia coli* ATCC 25922; and *Pseudomonas aeruginosa* ATCC 27583. The minimal inhibitory concentration (MIC; mg/L) was determined for each experimental sample.

No significant activity of compound **1** was observed under the conditions tested. The table with the obtained MIC values is included in the Supplementary Materials (Tables S7 and S8).

## 3. Materials and Methods

### 3.1. Synthesis

#### 3.1.1. Mechanochemical Synthesis

The metformin hydrochloride, maleic acid–oxalic acid dihydrate and all solvents were purchased commercially from EU and used as received.

Oxalic acid (β-polymorph) was gained by slow evaporation of water from a mixture of oxalic acid (α-polymorph) with its hydrate.

Compound **1**: An amount of 0.1 mmol of metformin hydrochloride was mixed with 2.5 mmol of oxalic acid dehydrate and 40 μL of H_2_O in a stainless-steel jar with steel balls of 3 mm in diameter. The LAG was performed in a ball mill operating at a 25 Hz frequency for an hour. The powder was washed with ethanol and acetone to eliminate unreacted substrates and by-products. The pure compound was left to dry, and the powder X-ray pattern was recorded.

Compound **2**: An amount of 0.1 mmol of metformin hydrochloride was mixed with 0.1 mmol of oxalic acid dehydrate and 20 μL of H_2_O in a stainless-steel jar with steel balls of 3 mm in diameter. The LAG was performed in a ball mill operating at a 25 Hz frequency for an hour. The powder was washed with methanol to eliminate unreacted substrates and by-products. The pure compound was left to dry, and the powder X-ray pattern was recorded.

Compound **3**: An amount of 0.1 mmol of metformin hydrochloride was mixed with 0.1 mmol of oxalic acid dehydrate and 20 μL of ethanol in a stainless-steel jar with steel balls of 3 mm in diameter. The LAG was performed in a ball mill operating at a 25 Hz frequency for an hour. The powder was washed with methanol to eliminate unreacted substrates and by-products. The pure compound was left to dry, and the powder X-ray pattern was recorded.

#### 3.1.2. Preparation of Single Crystals

Compound **1**: The powder obtained from the mechanochemical reaction was dissolved in DMSO and left for crystallization. After a few months, colorless crystals suitable for measurement were obtained.

Compounds **2** and **3**: An amount of 0.1 mmol of metformin hydrochloride was mixed with 0.1 mmol of oxalic acid dehydrate in DSMO and left for crystallization. After a few months, colorless crystals of both compounds, suitable for X-ray single crystal data collection, were obtained.

### 3.2. Powder X-Ray Measurement

Powder X-ray studies were performed with a Bruker D8 Quest (40 Manning Rd, Billerica, MA, USA) diffractometer with monochromated CuKα radiation (λ = 1.54178 A) at room temperature. The 2θ range for the measurement was 5–50° with a 120 s exposure time and a detector distance of 100 mm. The diffractograms were analyzed, and pictures were prepared with the KDif v.3.01b program [24].

### 3.3. Single-Crystal X-Ray Measurement and Refinement

Single-crystal X-ray diffraction data were collected on a Bruker D8 QUEST (40 Manning Rd, Billerica, MA, USA) diffractometer, equipped with a microfocus sealed tube (MoKα radiation, λ = 0.71073 Å (**1**, **2**) CuKα radiation λ = 1.54178 Å (**3**)), using a multilayer mirror as monochromator and a Bruker PHOTON III CPAD detector at 100(1) K for **1** and **3** and at room temperature for **2**. The data collection temperature was controlled by an Oxford Cryostream 600 low-temperature device (Oxford, UK). The data were integrated with SAINT V8.41 [25]. Multi-scan absorption correction using SADABS 2016/2 was applied [26]. Accurate unit-cell parameters were determined by a least-squares fit of the reflections of the highest intensity, which were chosen from the whole experiment. The calculations were mainly performed within the OLEX2 1.5 [27]. The structures were solved with ShelxT [28] and refined with the full-matrix least-squares procedure on F2 by SHELXL-2015/2017 [29]. Scattering factors incorporated in SHELXL were used. All atoms were refined anisotropically, HAR was performed using the NoSpherA2 procedure [30] implemented in Olex2 1.5 with the use of the ORCA 5.0/6.0 software [31] (r2SCAN method; basis set: cc-pVTZ). The structural representation was prepared with Mercury (ver. 2022.3.0) [32] and the MoPro Viewer software (v. 2018 and 2023) [33]. The crystallographic data and refinement details are listed in Table 4. The topological calculations and multipolar parameter transfer were performed using the MoPro software (v. 2018 and 2023) [33].

### 3.4. Microbiological Study

The microorganisms used in this study were obtained from ATCC (American Type Culture Collection, Manassas, VA, USA) or were acquired (MRSA, clinical strains) from the collection of the Department of Genetics and Pharmaceutical Microbiology at the Poznan University of Medical Sciences, Poland.

The minimal inhibitory concentration (MIC; mg/L) was determined for each experimental sample. The assessment was performed according to the methodology recommended by the European Society of Clinical Microbiology and Infectious Diseases (EUCAST), using the microdilution method with Mueller–Hinton broth (MHB; Oxoid, UK), under conditions suitable for the particular bacteria’s growth, using polystyrene plates.

Tested compounds were dissolved in DMSO (POCH, Gliwice, Poland) and diluted in sterile MHB to obtain the required concentration. The concentrations of the tested compounds in the liquid medium ranged from 64 to 0.03125 mg/L. The final inoculum of all studied organisms added to the serial dilution of assessed compounds was approximately 5.0 × 10^5^ CFU mL^−1^ (colony-forming units per mL).

Positive control samples (MHB with bacteria inoculation; without tested compounds) and sterility tests (MHB incubation without bacteria inoculation) were performed simultaneously. The samples were incubated for 18 h at 35–37 °C. After incubation, growth in the tested samples and the control samples was assessed visually.

## 4. Conclusions

This study presents a detailed analysis of multicomponent metformin crystals with oxalic and maleic acids, as well as chloride, obtained in different stoichiometric ratios. The crystal structures were determined by X-ray diffraction and further analyzed using the Atoms-in-Molecules (AIM) approach, focusing on critical points and the topology of electron density.

The CSD analysis showed that metformin in crystals usually occurs as a monoprotonated salt; diprotonated structures are definitely much less common. Among the latter, three types of structures were obtained: salt, cocrystal salt solvate and, previously not observed, mixed salt with both organic and inorganic anions. Protonation of the secondary imine nitrogen atoms in metformin affects bond lengths and electron density in the neighboring bonds. In the case of maleic acid salts, regardless of mono- or deprotonation of metformin, the counterion is hydrogenmaleate. The preference for this anion may be linked to the very strong intramolecular bonding present in this entity, caused by the molecule’s favorable geometry. Comparison with monoprotonated metformin reveals similar topological features despite different protonation states.

Mechanochemical synthesis using liquid-assisted grinding (LAG) allowed the selective formation of specific crystalline forms. The choice of solvent and the polymorphic form of the acids (α- or β-polymorph) significantly influenced the reaction outcome, although complete phase purity was not always achieved.

These findings highlight the importance of protonation, hydrogen bonding, and synthesis methodology in the design and control of multicomponent metformin crystal architectures.

## Figures and Tables

**Table 1:** Selected hydrogen-bond parameters for compound **1**.

D—H···A	D—H (Å)	H···A (Å)	D···A (Å)	D—H···A (°)
O31—H31···O15	0.98 (2)	1.54 (2)	2.5130 (11)	169.1 (15)
O21—H21···O26 ^i^	0.983 (19)	1.61 (2)	2.5904 (10)	175.4 (15)
O11—H11···O16 ^i^	0.99 (2)	1.56 (2)	2.5508 (12)	175.2 (14)
N6—H6···O26	1.031 (17)	1.685 (17)	2.7027 (12)	168.2 (13)
N5—H5b···O33 ^ii^	0.976 (16)	1.928 (16)	2.8987 (11)	172.6 (12)
N5—H5a···O16	1.009 (15)	1.842 (15)	2.8253 (12)	163.7 (12)
N9—H9a···O13 ^iii^	1.020 (15)	1.957 (16)	2.9676 (13)	170.4 (13)
N9—H9b···O31 ^iv^	0.978 (16)	2.157 (17)	3.0397 (13)	149.4 (14)
N9—H9b···O33 ^v^	0.978 (16)	2.323 (17)	3.1150 (12)	137.5 (13)
N8—H8a···O25 ^vi^	1.028 (16)	1.883 (17)	2.8520 (14)	155.8 (12)
N8—H8b···O1w ^vii^	1.025 (17)	1.847 (17)	2.8612 (14)	169.8 (12)
O1w—H1w···O33 ^viii^	0.955 (18)	2.261 (18)	3.1306 (13)	150.9 (16)
O1w—H2w···O25	0.986 (18)	1.818 (18)	2.8030 (12)	177.9 (16)

Symmetry codes: (i) *x* − 1, *y*, *z*; (ii) −*x* + 1, −*y* + 1, −*z* + 2; (iii) *x* + 1, *y*, *z*; (iv) *x*, *y* + 1, *z*; (v) −*x* + 2, −*y* + 1, −*z* + 2; (vi) −*x* + 1, −*y* + 2, −*z* + 1; (vii) −*x* + 2, −*y* + 2, −*z* + 1; (viii) *x*, *y* + 1, *z* − 1.

**Table 2:** Selected hydrogen-bond parameters for compound **2**.

D—H···A	D—H (Å)	H···A (Å)	D···A (Å)	D—H···A (°)
N6—H6···O11 ^i^	1.00 (2)	1.84 (2)	2.8249 (17)	170.7 (17)
N8—H8A···O21A	1.03 (3)	1.83 (3)	2.855 (7)	178.3 (17)
N8—H8A···O21B	1.03 (3)	1.80 (5)	2.81 (4)	166 (2)
N8—H8B···O13	1.02 (2)	1.78 (2)	2.786 (2)	166.1 (16)
N9—H9A···O23	1.03 (2)	1.77 (2)	2.768 (2)	162.1 (18)
N9—H9B···O11 ^i^	1.01 (2)	2.66 (2)	3.350 (2)	125.5 (14)
N9—H9B···O13 ^i^	1.01 (2)	1.83 (2)	2.834 (2)	173 (2)
N5—H5A···O17 ^ii^	1.03 (2)	1.95 (2)	2.948 (2)	165.1 (19)
N5—H5B···O27 ^iii^	1.02 (2)	1.78 (2)	2.800 (2)	172.0 (18)
O11—H11···O18	1.41 (3)	1.06 (3)	2.4648 (16)	174 (2)
O18—H11···O11	1.06 (3)	1.41 (3)	2.4648 (16)	174 (2)
O28—H28···O21A	1.10 (2)	1.37 (2)	2.458 (6)	174 (2)
O28—H28···O21B	1.10 (2)	1.37 (3)	2.43 (3)	160 (3)
O21A—H28···O28	1.37 (2)	1.10 (2)	2.458 (6)	174 (2)
O21B—H28···O28	1.37 (3)	1.10 (2)	2.43 (3)	160 (3)

Symmetry codes: (i) *x* + 1/2, −*y* + 3/2, *z* + 1/2; (ii) −*x* + 1/2, *y* + 1/2, −*z* + 1/2; (iii) −*x* + 1/2, *y* + 1/2, −*z* + 3/2.

**Table 3:** Selected hydrogen-bond parameters for compound **3**.

D—H···A	D—H (Å)	H···A (Å)	D···A (Å)	D—H···A (°)
N5—H5A···O13 ^i^	0.999 (16)	1.916 (16)	2.8789 (13)	160.9 (13)
O1w—H1W···Cl1	0.99 (2)	2.11 (2)	3.0960 (12)	177.6 (14)
N9—H9A···O17 ^ii^	1.009 (16)	1.870 (16)	2.8006 (13)	152.0 (13)
N8—H8b···O11	1.001 (17)	2.003 (18)	2.9916 (14)	168.8 (13)
N8—H8A···Cl1 ^iii^	1.017 (16)	2.316 (16)	3.2933 (11)	160.8 (14)
O11—H11···O18	1.34 (2)	1.10 (2)	2.4369 (11)	174.3 (16)
O18—H11···O11	1.10 (2)	1.34 (2)	2.4369 (11)	174.3 (16)
N5—H5B···O17 ^iv^	0.992 (17)	2.048 (18)	2.9642 (13)	152.6 (13)
O1w—H2W···Cl1 ^iii^	0.96 (2)	2.16 (2)	3.1025 (10)	164.4 (17)
N6—H6···O1w	1.04 (2)	1.64 (2)	2.6872 (12)	178.1 (15)
N9—H9b···O13	1.034 (18)	1.737 (18)	2.7597 (12)	169.3 (13)

Symmetry codes: (i) −*x* + 2, −*y*, −*z* + 1; (ii) *x*, *y* − 1, *z*; (iii) −*x* + 1, −*y* + 1, −*z*; (iv) −*x* + 2, −*y* + 1, −*z* + 1.

**Table 4:** Crystal data, data collection and structural refinement.

Compound	1	2	3
Formula	C_4_H_13_N_5_^2+^·2(C_2_HO_4_)^−^·1/2(C_2_H_2_O_4_)·H_2_O	C_4_H_13_N_5_^2+^·2(C_4_H_3_O_4_)^−^	C_4_H_13_N_5_^2+^·C_4_H_3_O_4_^−^· Cl^−^·H_2_O
Formula weight	372.27	361.31	299.71
Crystal system	Triclinic	Monoclinic	Triclinic
Space group	P-1	P2_1_/n	P-1
a (Å)	5.6975 (8)	8.2255 (8)	6.7749 (4)
b (Å)	9.8874 (9)	20.035 (2)	9.7279 (6)
c (Å)	14.7219 (16)	10.2794 (10)	11.6833 (7)
α (°)	88.026 (8)	90	67.583 (2)
β (°)	79.984 (6)	91.104 (6)	73.420 (2)
γ (°)	76.340 (6)	90	87.065 (2)
V (Å^3^)	793.58 (16)	1693.7 (3)	680.77 (7)
Z	2	4	2
μ (mm^−1^)	1.27	1.04	0.34
Reflections:			
Collected	10,379	16,577	30,649
Unique (Rint)	2888 (0.047)	3043 (0.048)	2581 (0.040)
With I > 2σ(I)	2493	2358	2460
R(F) [I > 2σ(I)]	0.022	0.035	0.022
wR(F^2^) [I > 2σ(I)]	0.048	0.062	0.088
Goodness of fit	1.09	1.07	1.15
max/min Δρ (e·Å^−3^)	0.29/−0.29	0.24, −0.24	0.41, −0.26
CCDC deposition number	2533107	2533108	2533109

## Data Availability

The original contributions presented in this study are included in the article/Supplementary Materials. Further inquiries can be directed to the corresponding authors.

## Supplementary Materials

The supporting information can be downloaded at https://www.mdpi.com/article/10.3390/ijms27073120/s1.

## References

[1] Marshall S.M. (2017). 60 Years of Metformin Use: A Glance at the Past and a Look to the Future. Diabetologia.

[2] Bailey C.J. (2017). Metformin: Historical Overview. Diabetologia.

[3] Rena G., Hardie D.G., Pearson E.R. (2017). The Mechanisms of Action of Metformin. Diabetologia.

[4] Foretz M., Guigas B., Bertrand L., Pollak M., Viollet B. (2014). Metformin: From Mechanisms of Action to Therapies. Cell Metab..

[5] Tsironikos G.I., Tsolaki V., Zakynthinos G.E., Rammou V., Kyprianidou D., Antonogiannis T., Zakynthinos E., Bargiota A. (2025). Metformin’s Overall Effectiveness and Combined Action with Lifestyle Interventions in Preventing Type-2 Diabetes Mellitus in High-Risk Metformin-Naïve Patients: An Updated Systematic Review and Meta-Analysis of Published RCTs. J. Clin. Med..

[6] Groom C.R., Bruno I.J., Lightfoot M.P., Ward S.C. (2016). The Cambridge Structural Database. Acta Crystallogr. Sect. B.

[7] (1971). Crystallography: Protein Data Bank. Nat. New Biol..

[8] Li P., Zhu Z., Wang Y., Zhang X., Yang C., Zhu Y., Zhou Z., Chao Y., Long Y., Gao Y. (2024). Substrate Transport and Drug Interaction of Human Thiamine Transporters SLC19A2/A3. Nat. Commun..

[9] Romane K., Peteani G., Mukherjee S., Kowal J., Rossi L., Hou J., Kossiakoff A.A., Lemmin T., Locher K.P. (2025). Structural Basis of Drug Recognition by Human MATE1 Transporter. Nat. Commun..

[10] Zhang S., Zhu A., Kong F., Chen J., Lan B., He G., Gao K., Cheng L., Sun X., Yan C. (2024). Structural Insights into Human Organic Cation Transporter 1 Transport and Inhibition. Cell Discov..

[11] Lu L.-P., Zhang H.-M., Feng S.-S., Zhu M.-L. (2004). Two N,N-Dimethylbiguanidium Salts Displaying Double Hydrogen Bonds to the Counter-Ions. Acta Crystallogr. Sect. C.

[12] Chong-Canto S., García-Báez E.V., Martínez-Martínez F.J., Ramos-Organillo A.A., Padilla-Martínez I.I. (2020). Mechanochemical Synthesis and Structure of the Tetrahydrate and Mesoporous Anhydrous Metforminium(2+)-N,N′-1,4-Phenylenedioxalamic Acid (1:2) Salt: The Role of Hydrogen Bonding and N→π* Charge Assisted Interactions. Pharmaceutics.

[13] Diniz L.F., Carvalho P.S., Gonçalves J.E., Diniz R., Fernandes C. (2022). Solid-State Landscape and Biopharmaceutical Implications of Novel Metformin-Based Salts. New J. Chem..

[14] Plata-Vargas E., de la Cruz-Hernández C., Dorazco-González A., Fuentes-Noriega I., Morales-Morales D., Germán-Acacio J.M. (2017). Synthesis of Metforminium Succinate by Melting. Crystal Structure, Thermal, Spectroscopic and Dissolution Properties. J. Mex. Chem. Soc..

[15] Grothe E., Meekes H., Vlieg E., ter Horst J.H., de Gelder R. (2016). Solvates, Salts, and Cocrystals: A Proposal for a Feasible Classification System. Cryst. Growth Des..

[16] Espinosa E., Molins E., Lecomte C. (1998). Hydrogen Bond Strengths Revealed by Topological Analyses of Experimentally Observed Electron Densities. Chem. Phys. Lett..

[17] Olar R., Badea M., Marinescu D.E. (2010). A new N,N-dimethylbiguanidium derivative-synthesis and structural characterization. Analele Univ. Bucur. Chim..

[18] Bader R.F.W. (1994). Atoms in Molecules: A Quantum Theory.

[19] Niranjana Devi R., Jelsch C., Israel S., Aubert E., Anzline C., Hosamani A.A. (2017). Charge Density Analysis of Metformin Chloride, a Biguanide Anti-Hyperglycemic Agent. Acta Crystallogr. Sect. B.

[20] Jayatilaka D., Dittrich B. (2008). X-Ray Structure Refinement Using Aspherical Atomic Density Functions Obtained from Quantum-Mechanical Calculations. Acta Crystallogr. Sect. A.

[21] Hansen N.K., Coppens P. (1978). Testing Aspherical Atom Refinements on Small-Molecule Data Sets. Acta Crystallogr. Sect. A.

[22] Domagała S., Fournier B., Liebschner D., Guillot B., Jelsch C. (2012). An Improved Experimental Databank of Transferable Multipolar Atom Models–ELMAM2. Construction Details and Applications. Acta Crystallogr. Sect. A.

[23] Munshi P., Row T.N.G. (2005). Charge Density Based Classification of Intermolecular Interactions in Molecular Crystals. CrystEngComm.

[24] Knizek K. Kalvados. https://www.fzu.cz/~knizek/kalvados/obr.html.

[25] Bruker A.X.S. (2024). SAINT, V8.41.

[26] Krause L., Herbst-Irmer R., Sheldrick G.M., Stalke D. (2015). Comparison of Silver and Molybdenum Microfocus X-Ray Sources for Single-Crystal Structure Determination. J. Appl. Crystallogr..

[27] Dolomanov O.V., Bourhis L.J., Gildea R.J., Howard J.A.K., Puschmann H. (2009). OLEX2: A Complete Structure Solution, Refinement and Analysis Program. J. Appl. Crystallogr..

[28] Sheldrick G.M. (2015). SHELXT–Integrated Space-Group and Crystal-Structure Determination. Acta Crystallogr. Sect. A.

[29] Sheldrick G.M. (2015). Crystal Structure Refinement with SHELXL. Acta Crystallogr. Sect. C.

[30] Kleemiss F., Dolomanov O.V., Bodensteiner M., Peyerimhoff N., Midgley L., Bourhis L.J., Genoni A., Malaspina L.A., Jayatilaka D., Spencer J.L. (2021). Accurate Crystal Structures and Chemical Properties from NoSpherA2. Chem. Sci..

[31] Neese F. (2012). The ORCA Program System. WIREs Comput. Mol. Sci..

[32] Macrae C.F., Sovago I., Cottrell S.J., Galek P.T.A., McCabe P., Pidcock E., Platings M., Shields G.P., Stevens J.S., Towler M. (2020). Mercury 4.0: From Visualization to Analysis, Design and Prediction. J. Appl. Crystallogr..

[33] Jelsch C., Guillot B., Lagoutte A., Lecomte C. (2005). Advances in Protein and Small-Molecule Charge-Density Refinement Methods Using MoPro. J. Appl. Crystallogr..

