# Supplementary material for: New Multicomponent Crystals of Antidiabetic Drug, Metformin: Mechanochemistry, Structural Studies, Biological Activity and Topological Analysis
Source: Int J Mol Sci. 2026 Mar 30;27(7):3120. doi: 10.3390/ijms27073120 (PMC13073212; doi:10.3390/ijms27073120)

checkCIF/PLATON report

Structure factors have been supplied for datablock(s) 1

THIS REPORT IS FOR GUIDANCE ONLY. IF USED AS PART OF A REVIEW PROCEDURE FOR PUBLICATION, IT SHOULD NOT REPLACE THE EXPERTISE OF AN EXPERIENCED CRYSTALLOGRAPHIC REFEREE.

No syntax errors found. CIF dictionary Interpreting this report

Datablock: 1

```
Bond precision:   C-C = 0.0020 A           Wavelength=1.54178

Cell:            a=5.6975(8)        b=9.8874(9)        c=14.7219(16)
                 alpha=88.026(8)    beta=79.984(6)     gamma=76.340(6)
Temperature:     100 K
```

	Calculated	Reported
Volume	793.58(16)	793.58(16)
Space group	P -1	P -1
Hall group	-P 1	-P 1
Moiety formula	C4 H13 N5, 2(C2 H O4), 0.5(C2 H2 O4), H2 O	C4 H13 N5, 2(C2 H O4), 0.5(C2 H2 O4), H2 O
Sum formula	C9 H18 N5 O11	C9 H18 N5 O11
Mr	372.28	372.28
Dx,g cm-3	1.558	1.558
Z	2	2
Mu (mm-1)	1.268	1.268
F000	390.0	390.0
F000’	391.64	
h,k,lmax	6,11,17	6,11,17
Nref	2921	2888
Tmin,Tmax		0.640,0.753
Tmin’		

```
Correction method= # Reported T Limits: Tmin=0.640 Tmax=0.753
AbsCorr = MULTI-SCAN
```

Data completeness= 0.989 Theta(max)= 68.674

R(reflections)= 0.0349(2493) wR2(reflections)= 0.0925(2888)

S = 1.170 Npar= 226

The following ALERTS were generated. Each ALERT has the format
test-name_ALERT_alert-type_alert-level.
Click on the hyperlinks for more details of the test.

Alert level C

```
PLAT055_ALERT_1_C Maximum Crystal Dimension Missing (or Error) ...     Please Check
PLAT218_ALERT_3_C Constrained U(i,j) Components(s) for H31                  6 Check
PLAT218_ALERT_3_C Constrained U(i,j) Components(s) for H21                  6 Check
PLAT218_ALERT_3_C Constrained U(i,j) Components(s) for H11                  6 Check
PLAT218_ALERT_3_C Constrained U(i,j) Components(s) for H6                   6 Check
PLAT218_ALERT_3_C Constrained U(i,j) Components(s) for H5B                  6 Check
PLAT218_ALERT_3_C Constrained U(i,j) Components(s) for H5A                  6 Check
PLAT218_ALERT_3_C Constrained U(i,j) Components(s) for H9A                  6 Check
PLAT218_ALERT_3_C Constrained U(i,j) Components(s) for H9B                  6 Check
PLAT218_ALERT_3_C Constrained U(i,j) Components(s) for H8A                  6 Check
PLAT218_ALERT_3_C Constrained U(i,j) Components(s) for H8B                  6 Check
PLAT218_ALERT_3_C Constrained U(i,j) Components(s) for H1B                  6 Check
PLAT218_ALERT_3_C Constrained U(i,j) Components(s) for H1C                  6 Check
PLAT218_ALERT_3_C Constrained U(i,j) Components(s) for H1A                  6 Check
PLAT218_ALERT_3_C Constrained U(i,j) Components(s) for H3B                  6 Check
PLAT218_ALERT_3_C Constrained U(i,j) Components(s) for H3C                  6 Check
PLAT218_ALERT_3_C Constrained U(i,j) Components(s) for H3A                  6 Check
PLAT218_ALERT_3_C Constrained U(i,j) Components(s) for H1W                  6 Check
PLAT218_ALERT_3_C Constrained U(i,j) Components(s) for H2W                  6 Check
PLAT250_ALERT_2_C Large U3/U1 Ratio for <U(i,j)> Tensor(Resd    2)        2.2 Note
PLAT353_ALERT_3_C Long   N-H (N0.87,N1.01A)  N5       - H5A      .       1.01 Ang.
PLAT353_ALERT_3_C Long   N-H (N0.87,N1.01A)  N6       - H6       .       1.03 Ang.
PLAT353_ALERT_3_C Long   N-H (N0.87,N1.01A)  N8       - H8A      .       1.03 Ang.
PLAT353_ALERT_3_C Long   N-H (N0.87,N1.01A)  N8       - H8B      .       1.03 Ang.
PLAT353_ALERT_3_C Long   N-H (N0.87,N1.01A)  N9       - H9A      .       1.02 Ang.
PLAT906_ALERT_3_C Large K Value in the Analysis of Variance ......      5.434 Check
PLAT911_ALERT_3_C Missing FCF Refl Between Thmin & STh/L=    0.600         17 Report
                1 -8  2,   1  2  2,   1  3  2,   2 -7  3,   1 -3  3,   0 -2  3,
                0-10  4,   0-11  5,   0 -8  5,   3 -8  5,   4 -7  5,   4 -7  6,
                3 -8  7,   3 -8  8,   4 -1  9,   3 -7 10,   1 -8 12,
```

Alert level G

```
PLAT007_ALERT_5_G Number of Unrefined Donor-H Atoms .............          12 Report
              H5A     H5B     H6      H8A     H8B     H9A     H9B     H11
              H21     H31     H1W     H2W
PLAT169_ALERT_4_G The CIF-Embedded .res File Contains AFIX 1 Recds          9 Report
PLAT186_ALERT_4_G The CIF-Embedded .res File Contains ISOR Records          2 Report
PLAT432_ALERT_2_G Short Inter X...Y Contact  O11      ..C32      .       2.88 Ang.
                                               -1+x,y,z  =      1_455 Check
PLAT432_ALERT_2_G Short Inter X...Y Contact  O15      ..C32      .       2.85 Ang.
                                             1-x,-y,2-z  =      2_657 Check
```

```
PLAT432_ALERT_2_G Short Inter X...Y Contact  C12       ..C12       .        3.14 Ang.
                                              -x,1-y,2-z  =      2_567 Check
PLAT432_ALERT_2_G Short Inter X...Y Contact  C24       ..C24       .        3.16 Ang.
                                             1-x,2-y,1-z  =      2_676 Check
PLAT860_ALERT_3_G Number of Least-Squares Restraints .............         12 Note
PLAT912_ALERT_4_G Missing # of FCF Reflections Above STh/L=  0.600         16 Note
PLAT941_ALERT_3_G Average HKL Measurement Multiplicity ..........         3.6 Low
PLAT969_ALERT_5_G The 'Henn et al.' R-Factor-gap value ..........       2.257 Note
             Predicted wR2: Based on SigI**2  4.10 or SHELX Weight  7.90
PLAT978_ALERT_2_G Number C-C Bonds with Positive Residual Density.          2 Info
PLAT994_ALERT_1_G SHELXL .ins Contains no or MERG 0 Instruction ..          ! Note
```

```
   0 ALERT level A = Most likely a serious problem - resolve or explain
   0 ALERT level B = A potentially serious problem, consider carefully
  27 ALERT level C = Check. Ensure it is not caused by an omission or oversight
  13 ALERT level G = General information/check it is not something unexpected

   2 ALERT type 1 CIF construction/syntax error, inconsistent or missing data
   6 ALERT type 2 Indicator that the structure model may be wrong or deficient
  27 ALERT type 3 Indicator that the structure quality may be low
   3 ALERT type 4 Improvement, methodology, query or suggestion
   2 ALERT type 5 Informative message, check
```

It is advisable to attempt to resolve as many as possible of the alerts in all categories. Often the minor alerts point to easily fixed oversights, errors and omissions in your CIF or refinement strategy, so attention to these fine details can be worthwhile. It is up to the individual to critically assess their own results and, if necessary, seek expert advice.

PLATON version of 15/01/2026; check.def file version of 02/01/2026

---

duplicate check

No duplication found

Datablock 1 - ellipsoid plot

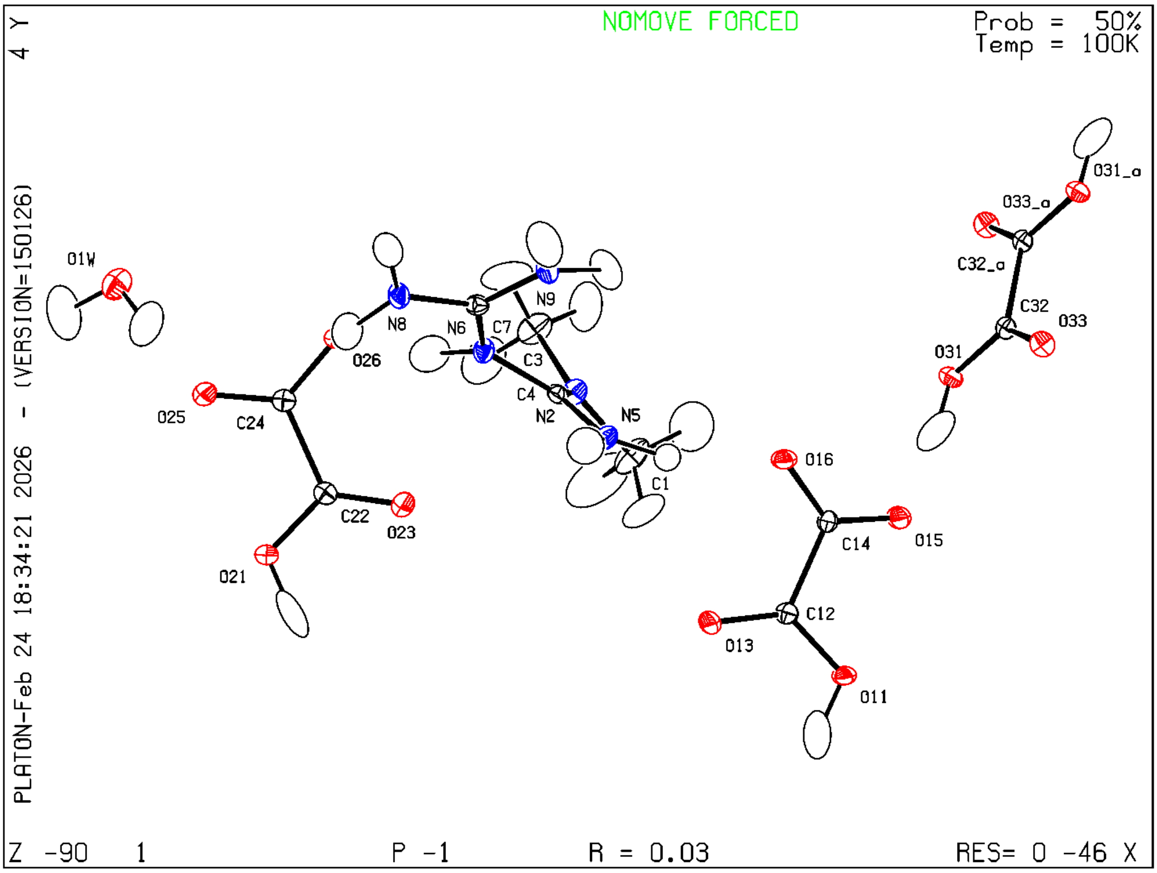

Supplement: Supplementary file 1 [file ijms-27-03120-s001.zip › 1_checkcif.pdf]
