# Supplementary material for: New Multicomponent Crystals of Antidiabetic Drug, Metformin: Mechanochemistry, Structural Studies, Biological Activity and Topological Analysis
Source: Int J Mol Sci. 2026 Mar 30;27(7):3120. doi: 10.3390/ijms27073120 (PMC13073212; doi:10.3390/ijms27073120)

checkCIF/PLATON report

Structure factors have been supplied for datablock(s) 261_3

THIS REPORT IS FOR GUIDANCE ONLY. IF USED AS PART OF A REVIEW PROCEDURE FOR PUBLICATION, IT SHOULD NOT REPLACE THE EXPERTISE OF AN EXPERIENCED CRYSTALLOGRAPHIC REFEREE.

No syntax errors found. CIF dictionary Interpreting this report

Datablock: 261_3

Bond precision:	C-C = 0.0023 A		Wavelength=1.54178
Cell:	a=8.2255(8)	b=20.035(2)	c=10.2794(10)
	alpha=90	beta=91.104(6)	gamma=90
Temperature:	291 K		

	Calculated	Reported
Volume	1693.7(3)	1693.7(3)
Space group	P 21/n	P 1 21/n 1
Hall group	-P 2yn	-P 2yn
Moiety formula	2(C4 H3 O4), C4 H13 N5	2(C4 H3 O4), C4 H13 N5
Sum formula	C12 H19 N5 O8	C12 H19 N5 O8
Mr	361.32	361.31
Dx,g cm-3	1.417	1.417
Z	4	4
Mu (mm-1)	1.036	1.039
F000	760.0	760.0
F000'	762.90	
h,k,lmax	9,24,12	9,24,12
Nref	3097	3043
Tmin,Tmax	0.829,0.901	0.692,0.753
Tmin'	0.771	

Correction method= # Reported T Limits: Tmin=0.692 Tmax=0.753
AbsCorr = MULTI-SCAN

Data completeness= 0.983   Theta(max)= 68.320

R(reflections)= 0.0349(2358)   wR2(reflections)= 0.0624(3043)

S = 1.066   Npar= 407

The following ALERTS were generated. Each ALERT has the format
test-name_ALERT_alert-type_alert-level.
Click on the hyperlinks for more details of the test.

Alert level B

```
PLAT088_ALERT_3_B Poor Data / Parameter Ratio ....................       7.48 Note
```

Alert level C

```
PLAT250_ALERT_2_C Large U3/U1 Ratio for <U(i,j)> Tensor(Resd    1)        2.4 Note
PLAT250_ALERT_2_C Large U3/U1 Ratio for <U(i,j)> Tensor(Resd    3)        3.8 Note
PLAT303_ALERT_2_C Full Occupancy Atom H28       with # Connections       2.00 Check
PLAT776_ALERT_1_C Check   D-H Dist in CIF: O11      --H11        ..      1.41 Ang.
PLAT776_ALERT_1_C Check   D-H Dist in CIF: O21A     --H28        ..      1.37 Ang.
PLAT776_ALERT_1_C Check   D-H Dist in CIF: O21B     --H28        ..      1.37 Ang.
PLAT906_ALERT_3_C Large K Value in the Analysis of Variance ......      2.894 Check
PLAT911_ALERT_3_C Missing FCF Refl Between Thmin & STh/L=    0.600         39 Report
                 9  0  1,   9  1  1,   1  2  1,  -1  3  1,  -2  1  2,  -8  5  2,
                 0  6  2,   8 10  2,  -7  4  3,  -8  5  3,  -8  1  4,  -9  0  5,
                -8  4  5,  -7  4  5,  -8  0  6,  -8  5  6,  -3  0  7,  -3  1  7,
                -4  0  8,  -4  1  8,  -6 11  8,  -5  0  9,  -6  1  9,  -5  1  9,
                -4  1  9,  -6  2  9,  -5  2  9,  -4  2  9,  -5  1 10,  -5  2 10,
                    (    9 More NOT listed: see .ckf listing file)
```

Alert level G

```
PLAT002_ALERT_2_G Number of Distance or Angle Restraints on AtSite          4 Note
PLAT176_ALERT_4_G The CIF-Embedded .res File Contains SADI Records          2 Report
PLAT300_ALERT_4_G Atom Site Occupancy of O21A       Constrained at       0.85 Check
PLAT300_ALERT_4_G Atom Site Occupancy of O21B       Constrained at       0.15 Check
PLAT301_ALERT_3_G Main Residue  Disorder ..............(Resd    1)        13% Note
PLAT303_ALERT_2_G Full Occupancy Atom H11       with # Connections       2.00 Check
PLAT480_ALERT_4_G Long H...A H-Bond Reported H9B      ..O11       .      2.66 Ang.
PLAT778_ALERT_2_G Check O..H..X Bond in CIF: O11      --H11              1.41 Ang.
PLAT778_ALERT_2_G Check O..H..X Bond in CIF: O21A     --H28              1.37 Ang.
PLAT778_ALERT_2_G Check O..H..X Bond in CIF: H28      --O21B             1.37 Ang.
PLAT779_ALERT_4_G Suspect or Irrelevant (Bond) Angle(s) in CIF ...      19.40 Deg.
              O21B -C22  -O21A    1_555   1_555   1_555 ......     #   44 Check
PLAT779_ALERT_4_G Suspect or Irrelevant (Bond) Angle(s) in CIF ...      18.20 Deg.
              O21B -H28  -O21A    1_555   1_555   1_555 ......     #   68 Check
PLAT802_ALERT_4_G CIF Input Record(s) with more than 80 Characters          1 Info
PLAT860_ALERT_3_G Number of Least-Squares Restraints .............          4 Note
PLAT883_ALERT_1_G Absent Datum for _atom_sites_solution_primary ..     Please Do !
PLAT912_ALERT_4_G Missing # of FCF Reflections Above STh/L=  0.600         15 Note
PLAT969_ALERT_5_G The 'Henn et al.' R-Factor-gap value ...........      1.932 Note
              Predicted wR2: Based on SigI**2  3.22 or SHELX Weight  5.84
PLAT978_ALERT_2_G Number C-C Bonds with Positive Residual Density.          6 Info
```

```
PLAT979_ALERT_1_G NoSpherA2 Scattering Factors Used .............     Please Note
PLAT994_ALERT_1_G SHELXL .ins Contains no or MERG 0 Instruction ..         ! Note
```

```
   0 ALERT level A = Most likely a serious problem - resolve or explain
   1 ALERT level B = A potentially serious problem, consider carefully
   8 ALERT level C = Check. Ensure it is not caused by an omission or oversight
  20 ALERT level G = General information/check it is not something unexpected

   6 ALERT type 1 CIF construction/syntax error, inconsistent or missing data
   9 ALERT type 2 Indicator that the structure model may be wrong or deficient
   5 ALERT type 3 Indicator that the structure quality may be low
   8 ALERT type 4 Improvement, methodology, query or suggestion
   1 ALERT type 5 Informative message, check
```

It is advisable to attempt to resolve as many as possible of the alerts in all categories. Often the minor alerts point to easily fixed oversights, errors and omissions in your CIF or refinement strategy, so attention to these fine details can be worthwhile. It is up to the individual to critically assess their own results and, if necessary, seek expert advice.

PLATON version of 15/01/2026; check.def file version of 02/01/2026

---

duplicate check

No duplication found

Datablock 261_3 - ellipsoid plot

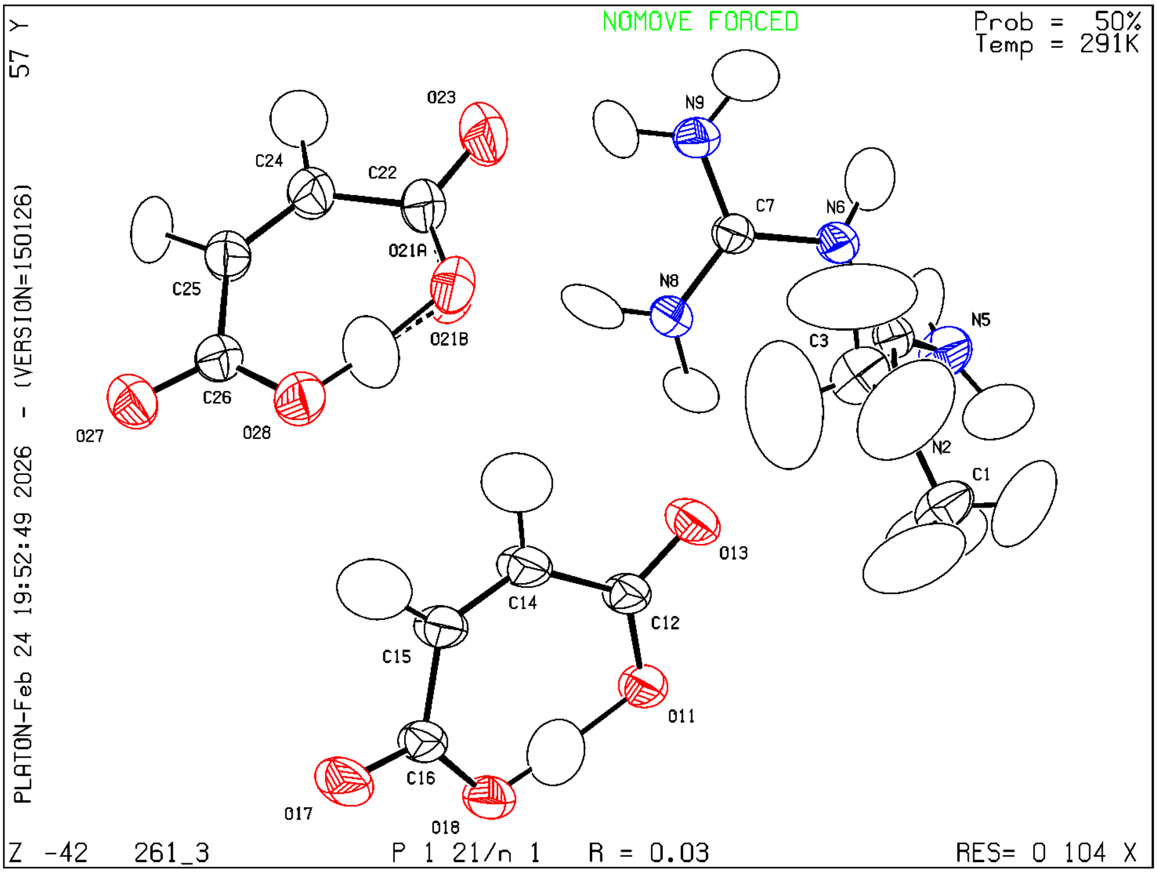

Supplement: Supplementary file 1 [file ijms-27-03120-s001.zip › 2_checkcif.pdf]
