# Supplementary material for: New Multicomponent Crystals of Antidiabetic Drug, Metformin: Mechanochemistry, Structural Studies, Biological Activity and Topological Analysis
Source: Int J Mol Sci. 2026 Mar 30;27(7):3120. doi: 10.3390/ijms27073120 (PMC13073212; doi:10.3390/ijms27073120)

checkCIF/PLATON report

Structure factors have been supplied for datablock(s) 3

THIS REPORT IS FOR GUIDANCE ONLY. IF USED AS PART OF A REVIEW PROCEDURE FOR PUBLICATION, IT SHOULD NOT REPLACE THE EXPERTISE OF AN EXPERIENCED CRYSTALLOGRAPHIC REFEREE.

No syntax errors found. CIF dictionary Interpreting this report

Datablock: 3

Bond precision:	C-C = 0.0030 A		Wavelength=0.71073
Cell:	a=6.7749(4)	b=9.7279(6)	c=11.6833(7)
	alpha=67.583(2)	beta=73.420(2)	gamma=87.065(2)
Temperature:	273 K		

	Calculated	Reported
Volume	680.77(7)	680.77(7)
Space group	P -1	P -1
Hall group	-P 1	-P 1
Moiety formula	C4 H13 N5, C4 H3 O4, Cl, H2 O	?
Sum formula	C8 H18 Cl N5 O5	C8 H18 Cl N5 O5
Mr	299.72	299.72
Dx,g cm-3	1.462	1.462
Z	2	2
Mu (mm-1)	0.306	0.306
F000	316.0	316.0
F000’	316.44	
h,k,lmax	8,11,14	8,11,14
Nref	2590	2581
Tmin,Tmax	0.957,0.970	0.682,0.746
Tmin’	0.935	

Correction method= # Reported T Limits: Tmin=0.682 Tmax=0.746
AbsCorr = MULTI-SCAN

Data completeness= 0.997 Theta(max)= 25.680

R(reflections)= 0.0369(2460) wR2(reflections)= 0.1199(2581)

S = 1.108 Npar= 172

The following ALERTS were generated. Each ALERT has the format
test-name_ALERT_alert-type_alert-level.
Click on the hyperlinks for more details of the test.

Alert level B

```
PLAT934_ALERT_3_B Number of (Iobs-Icalc)/Sigma(W) > 10 Outliers ..          2 Check
                8 -1  2,   8  2  4,
```

Alert level C

```
PLAT218_ALERT_3_C Constrained U(i,j) Components(s) for H5A                  6 Check
PLAT218_ALERT_3_C Constrained U(i,j) Components(s) for H14                  6 Check
PLAT218_ALERT_3_C Constrained U(i,j) Components(s) for H1WA                 6 Check
PLAT218_ALERT_3_C Constrained U(i,j) Components(s) for H9A                  6 Check
PLAT218_ALERT_3_C Constrained U(i,j) Components(s) for H15                  6 Check
PLAT218_ALERT_3_C Constrained U(i,j) Components(s) for H3A                  6 Check
PLAT218_ALERT_3_C Constrained U(i,j) Components(s) for H1A                  6 Check
PLAT218_ALERT_3_C Constrained U(i,j) Components(s) for H3B                  6 Check
PLAT218_ALERT_3_C Constrained U(i,j) Components(s) for H3C                  6 Check
PLAT218_ALERT_3_C Constrained U(i,j) Components(s) for H1B                  6 Check
PLAT218_ALERT_3_C Constrained U(i,j) Components(s) for H8A                  6 Check
PLAT218_ALERT_3_C Constrained U(i,j) Components(s) for H8B                  6 Check
PLAT218_ALERT_3_C Constrained U(i,j) Components(s) for H1C                  6 Check
PLAT218_ALERT_3_C Constrained U(i,j) Components(s) for H11                  6 Check
PLAT218_ALERT_3_C Constrained U(i,j) Components(s) for H5B                  6 Check
PLAT218_ALERT_3_C Constrained U(i,j) Components(s) for H1WB                 6 Check
PLAT218_ALERT_3_C Constrained U(i,j) Components(s) for H6                   6 Check
PLAT218_ALERT_3_C Constrained U(i,j) Components(s) for H9B                  6 Check
PLAT353_ALERT_3_C Long   N-H (N0.87,N1.01A)  N6       - H6       .       1.04 Ang.
PLAT353_ALERT_3_C Long   N-H (N0.87,N1.01A)  N8       - H8B      .       1.02 Ang.
PLAT353_ALERT_3_C Long   N-H (N0.87,N1.01A)  N9       - H9A      .       1.01 Ang.
PLAT353_ALERT_3_C Long   N-H (N0.87,N1.01A)  N9       - H9B      .       1.03 Ang.
PLAT417_ALERT_2_C Short Inter D-H..H-D       H1WB     ..H6       .       2.11 Ang.
                                                      x,y,z  =      1_555 Check
PLAT911_ALERT_3_C Missing FCF Refl Between Thmin & STh/L=    0.600          8 Report
               -6  7  1,   7 -5  2,   7 -4  2,   7 -2  2,   7  2  3,   2 -6  4,
                7  6  5,   1  9  5,
```

Alert level G

```
PLAT007_ALERT_5_G Number of Unrefined Donor-H Atoms .............          10 Report
              H5A     H5B     H6      H8A     H8B     H9A     H9B     H11
              H1WA    H1WB
PLAT154_ALERT_1_G The s.u.'s on the Cell Angles are Equal ..(Note)      0.002 Degree
PLAT169_ALERT_4_G The CIF-Embedded .res File Contains AFIX 1 Recds         18 Report
PLAT186_ALERT_4_G The CIF-Embedded .res File Contains ISOR Records          2 Report
PLAT199_ALERT_1_G Reported _cell_measurement_temperature ..... (K)        273 Check
```

```
PLAT200_ALERT_1_G Reported   _diffrn_ambient_temperature ..... (K)        273 Check
PLAT303_ALERT_2_G Full Occupancy Atom H11       with # Connections       2.00 Check
PLAT720_ALERT_4_G Number of Unusual/Non-Standard Labels .........           2 Note
              H1WA    H1WB
PLAT778_ALERT_2_G Check O..H..X Bond in CIF: O11      --H11              1.34 Ang.
PLAT860_ALERT_3_G Number of Least-Squares Restraints ............          18 Note
PLAT910_ALERT_3_G Missing FCF Reflection(s) Below Theta(Min)[Deg]=       2.27 Note
                0  0  1,
PLAT912_ALERT_4_G Missing # of FCF Reflections Above STh/L=  0.600          1 Note
PLAT969_ALERT_5_G The 'Henn et al.' R-Factor-gap value ..........      6.237 Note
              Predicted wR2: Based on SigI**2  1.92 or SHELX Weight 10.82
PLAT978_ALERT_2_G Number C-C Bonds with Positive Residual Density.          1 Info
PLAT994_ALERT_1_G SHELXL .ins Contains no or MERG 0 Instruction ..          ! Note
```

```
   0 ALERT level A = Most likely a serious problem - resolve or explain
   1 ALERT level B = A potentially serious problem, consider carefully
  24 ALERT level C = Check. Ensure it is not caused by an omission or oversight
  15 ALERT level G = General information/check it is not something unexpected

   4 ALERT type 1 CIF construction/syntax error, inconsistent or missing data
   4 ALERT type 2 Indicator that the structure model may be wrong or deficient
  26 ALERT type 3 Indicator that the structure quality may be low
   4 ALERT type 4 Improvement, methodology, query or suggestion
   2 ALERT type 5 Informative message, check
```

It is advisable to attempt to resolve as many as possible of the alerts in all categories. Often the minor alerts point to easily fixed oversights, errors and omissions in your CIF or refinement strategy, so attention to these fine details can be worthwhile. It is up to the individual to critically assess their own results and, if necessary, seek expert advice.

PLATON version of 15/01/2026; check.def file version of 02/01/2026

---

duplicate check

No duplication found

Datablock 3 - ellipsoid plot

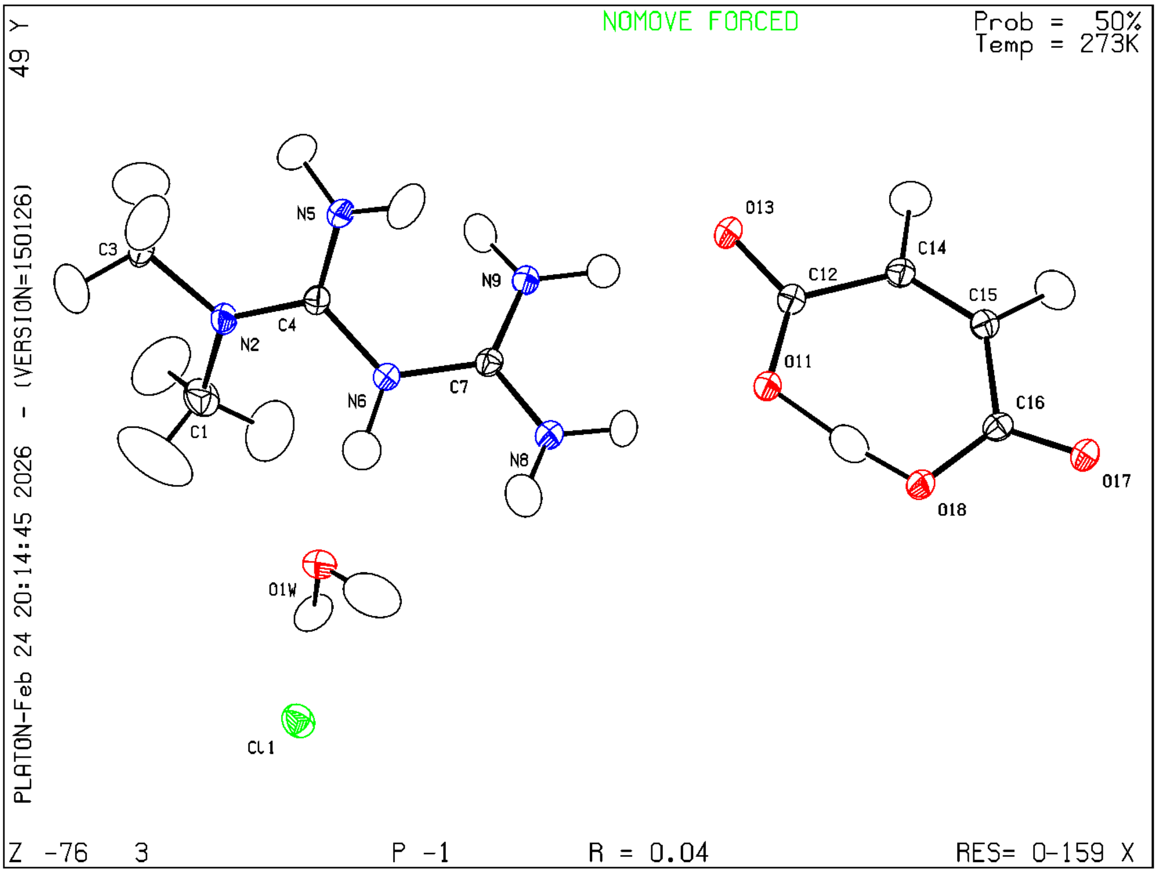

Supplement: Supplementary file 1 [file ijms-27-03120-s001.zip › 3_checkcif.pdf]
