# Supplementary material for: New Multicomponent Crystals of Antidiabetic Drug, Metformin: Mechanochemistry, Structural Studies, Biological Activity and Topological Analysis
Source: Int J Mol Sci. 2026 Mar 30;27(7):3120. doi: 10.3390/ijms27073120 (PMC13073212; doi:10.3390/ijms27073120)
Supplement: Supplementary file 1 [file ijms-27-03120-s001.zip › supll metf _2.pdf]

Supplementary materials

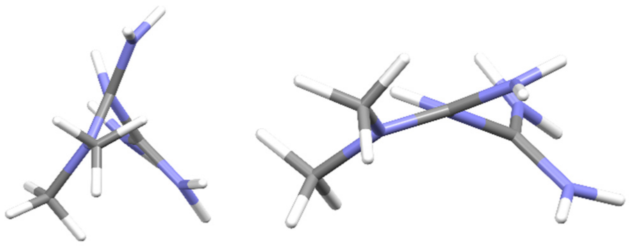

Figure S1. Two metformin conformers (left) type I, (right) type II. The view along C4-N6 bond.

Table S1. Geometric parameters for compound **1** (Å, º).

O31—C32	1.2931 (12)	N6—C7	1.3680 (13)
O33—C32	1.2181 (11)	N5—C4	1.3173 (12)
O21—C22	1.3099 (13)	N2—C4	1.3236 (12)
O11—C12	1.2981 (12)	N2—C1	1.4569 (13)
O26—C24	1.2628 (12)	N2—C3	1.4590 (13)
O15—C14	1.2489 (12)	N9—C7	1.3197 (13)
O13—C12	1.2145 (12)	N8—C7	1.3185 (14)
O16—C14	1.2512 (11)	C14—C12	1.5490 (14)
O25—C24	1.2406 (12)	C32—C32^{i}	1.5447 (19)
O23—C22	1.2121 (12)	C22—C24	1.5530 (13)
N6—C4	1.3803 (12)		
C7—N6—C4	125.28 (9)	O13—C12—O11	126.45 (10)
C1—N2—C4	122.11 (8)	C14—C12—O11	112.50 (9)
C3—N2—C4	122.82 (9)	C14—C12—O13	121.05 (8)
C3—N2—C1	115.01 (9)	O33—C32—O31	127.34 (9)
O16—C14—O15	127.52 (9)	C32^{i}—C32—O31	112.12 (9)
C12—C14—O15	116.69 (8)	C32^{i}—C32—O33	120.54 (10)
C12—C14—O16	115.78 (8)	O23—C22—O21	125.97 (9)
N5—C4—N6	119.70 (9)	C24—C22—O21	112.17 (8)
N2—C4—N6	117.48 (8)	C24—C22—O23	121.83 (9)
N2—C4—N5	122.76 (9)	O25—C24—O26	126.39 (9)
N9—C7—N6	120.11 (10)	C22—C24—O26	115.43 (8)
N8—C7—N6	117.80 (9)	C22—C24—O25	118.18 (9)
N8—C7—N9	122.06 (10)		
O31—C32—C32^{i}—O31^{i}	180.00 (11)	O13—C12—C14—O16	17.75 (10)

O31—C32—C32^{i}—O33^{i}	-0.01 (8)	O25—C24—C22—O23	175.21 (9)
O33—C32—C32^{i}—O31^{i}	0.01 (9)	N6—C4—N2—C1	-159.76 (10)
O33—C32—C32^{i}—O33^{i}	-180.00 (12)	N6—C4—N2—C3	17.41 (13)
O21—C22—C24—O26	177.82 (8)	N5—C4—N6—C7	39.79 (12)
O21—C22—C24—O25	-3.13 (9)	N5—C4—N2—C1	17.41 (13)
O11—C12—C14—O15	18.86 (9)	N5—C4—N2—C3	-165.42 (11)
O11—C12—C14—O16	-161.76 (8)	N2—C4—N6—C7	-142.96 (9)
O26—C24—C22—O23	-3.84 (10)	N9—C7—N6—C4	26.85 (11)
O15—C14—C12—O13	-161.64 (8)	N8—C7—N6—C4	-155.15 (9)

Table S2. Geometric parameters for compound **2** (Å, º)

O11—C12	1.2597 (17)	N9—C7	1.3187 (19)
O18—C16	1.2890 (19)	N5—C4	1.322 (2)
O27—C26	1.2266 (19)	O23—C22	1.2375 (19)
O28—C26	1.293 (2)	C26—C25	1.484 (2)
N6—C7	1.3644 (19)	C12—C14	1.489 (2)
N6—C4	1.3891 (19)	C16—C15	1.484 (2)
O13—C12	1.2445 (17)	C16—O17	1.2233 (18)
O21A—C22	1.275 (7)	C25—C24	1.338 (2)
N8—C7	1.3164 (19)	C24—C22	1.489 (3)
N2—C4	1.3149 (18)	C22—O21B	1.29 (3)
N2—C3	1.459 (2)	C15—C14	1.346 (2)
N2—C1	1.462 (2)		
C4—N6—C7	125.65 (13)	O17—C16—O18	120.95 (15)
C3—N2—C4	123.22 (15)	O17—C16—C15	119.03 (15)
C1—N2—C4	120.18 (15)	C24—C25—C26	130.94 (18)
C1—N2—C3	116.29 (17)	C22—C24—C25	130.51 (18)
N8—C7—N6	121.98 (14)	O23—C22—O21A	123.4 (3)
N9—C7—N6	117.03 (14)	C24—C22—O21A	120.8 (3)
N9—C7—N8	120.98 (15)	C24—C22—O23	115.80 (17)
N2—C4—N6	120.52 (13)	O21B—C22—O21A	19.4 (15)
N5—C4—N6	115.93 (15)	O21B—C22—O23	125.9 (17)
N5—C4—N2	123.52 (15)	O21B—C22—C24	115.2 (17)
O28—C26—O27	121.68 (16)	C14—C15—C16	130.87 (17)
C25—C26—O27	117.84 (16)	C15—C14—C12	130.79 (16)
C25—C26—O28	120.47 (15)	O18—H11—O11	174 (2)
O13—C12—O11	122.69 (14)	O21A—H28—O28	174 (2)
C14—C12—O11	120.06 (14)	O21B—H28—O28	160 (3)
C14—C12—O13	117.24 (14)	O21B—H28—O21A	18.2 (14)
C15—C16—O18	120.03 (14)		

O11—C12—C14—C15	3.2 (2)	N9—C7—N6—C4	172.23 (14)
O18—C16—C15—C14	-8.2 (2)	N5—C4—N6—C7	126.76 (15)
O27—C26—C25—C24	-178.17 (16)	N5—C4—N2—C3	164.6 (2)
O28—C26—C25—C24	2.4 (2)	N5—C4—N2—C1	-8.8 (2)
N6—C4—N2—C3	-13.4 (2)	O23—C22—C24—C25	179.39 (17)
N6—C4—N2—C1	173.20 (18)	C26—C25—C24—C22	1.5 (3)
O13—C12—C14—C15	-177.5 (2)	C12—C14—C15—C16	1.4 (3)
O21A—C22—C24—C25	-2.9 (3)	C25—C24—C22—O21B	18.2 (14)
N8—C7—N6—C4	-8.9 (2)	C14—C15—C16—O17	171.9 (3)
N2—C4—N6—C7	-55.14 (18)		

Table S3. Geometric parameters for compound **3** (Å, º)

O11—C12	1.2754 (13)	N2—C4	1.3263 (14)
O13—C12	1.2459 (13)	N2—C3	1.4594 (14)
O17—C16	1.2350 (13)	N2—C1	1.4598 (15)
O18—C16	1.2934 (13)	N8—C7	1.3212 (14)
N6—C7	1.3699 (14)	C16—C15	1.4869 (14)
N6—C4	1.3726 (13)	C14—C12	1.4951 (15)
N9—C7	1.3128 (14)	C14—C15	1.3423 (16)
N5—C4	1.3225 (14)		
C4—N6—C7	124.62 (9)	O18—C16—O17	121.71 (9)
C3—N2—C4	120.16 (10)	C15—C16—O17	117.70 (10)
C1—N2—C4	122.46 (10)	C15—C16—O18	120.58 (9)
C1—N2—C3	116.53 (10)	C15—C14—C12	130.42 (10)
N9—C7—N6	120.82 (10)	O13—C12—O11	123.13 (10)
N8—C7—N6	118.04 (10)	C14—C12—O11	120.61 (9)
N8—C7—N9	121.09 (11)	C14—C12—O13	116.26 (9)
N5—C4—N6	121.10 (10)	C14—C15—C16	130.05 (10)
N2—C4—N6	117.43 (9)	O18—H11—O11	174.3 (16)
N2—C4—N5	121.39 (10)		
O11—C12—C14—C15	-4.28 (13)	N5—C4—N6—C7	-29.44 (12)
O13—C12—C14—C15	176.11 (10)	N5—C4—N2—C3	-8.81 (12)
O17—C16—C15—C14	-173.49 (10)	N5—C4—N2—C1	160.29 (10)
O18—C16—C15—C14	7.58 (13)	N2—C4—N6—C7	153.84 (9)
N6—C4—N2—C3	167.89 (9)	N8—C7—N6—C4	153.27 (9)
N6—C4—N2—C1	-23.00 (12)	C16—C15—C14—C12	0.85 (15)
N9—C7—N6—C4	-29.30 (12)		

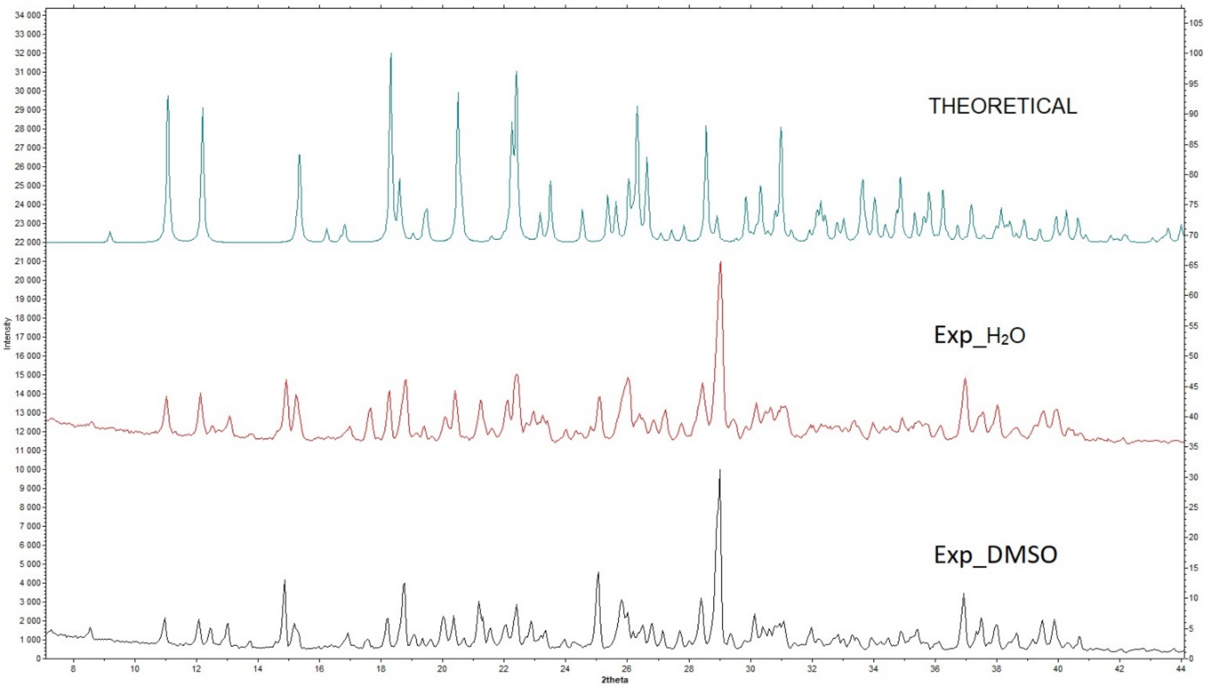

Figure S2. The PXR diffractogram of compound **1** prepared in different solvents in comparison to theoretical one generated from the structure of **1**

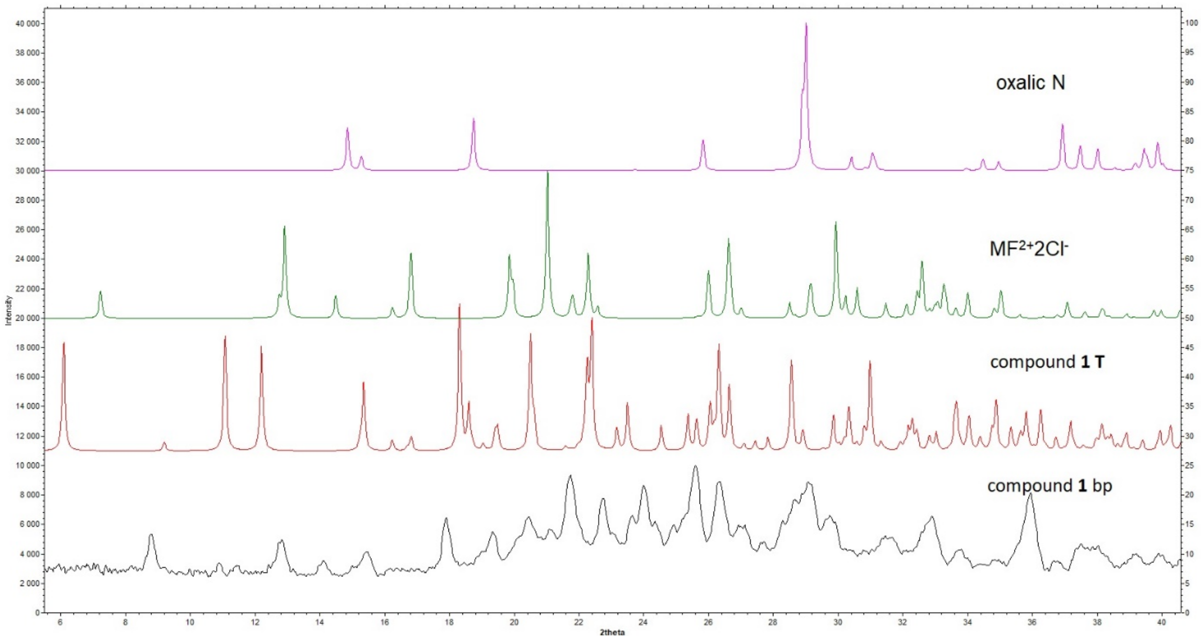

Figure S3. The PXR diffractogram of the results of the mechanochemistry reaction with few drops of DMSO between MfCl and oxalic acid in beta form - compound **1 bp** in comparison to theoretical one of: **1** - compound **1** T , $\mathbf{MF^{2+}2Cl^{-}}$ and oxalic acid hydrate – oxalic N

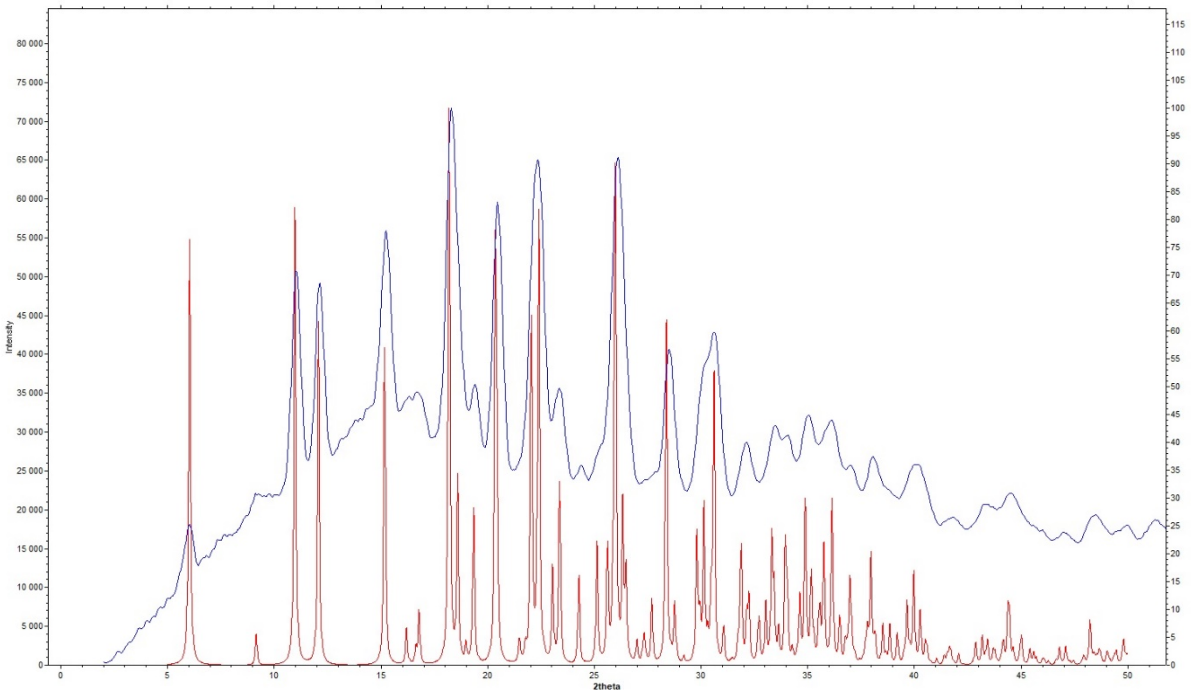

Figure S4. The PXR diffractogram of compound **1**; the results of the mechanochemistry reaction after the purification (blue experimental, red theoretical one)

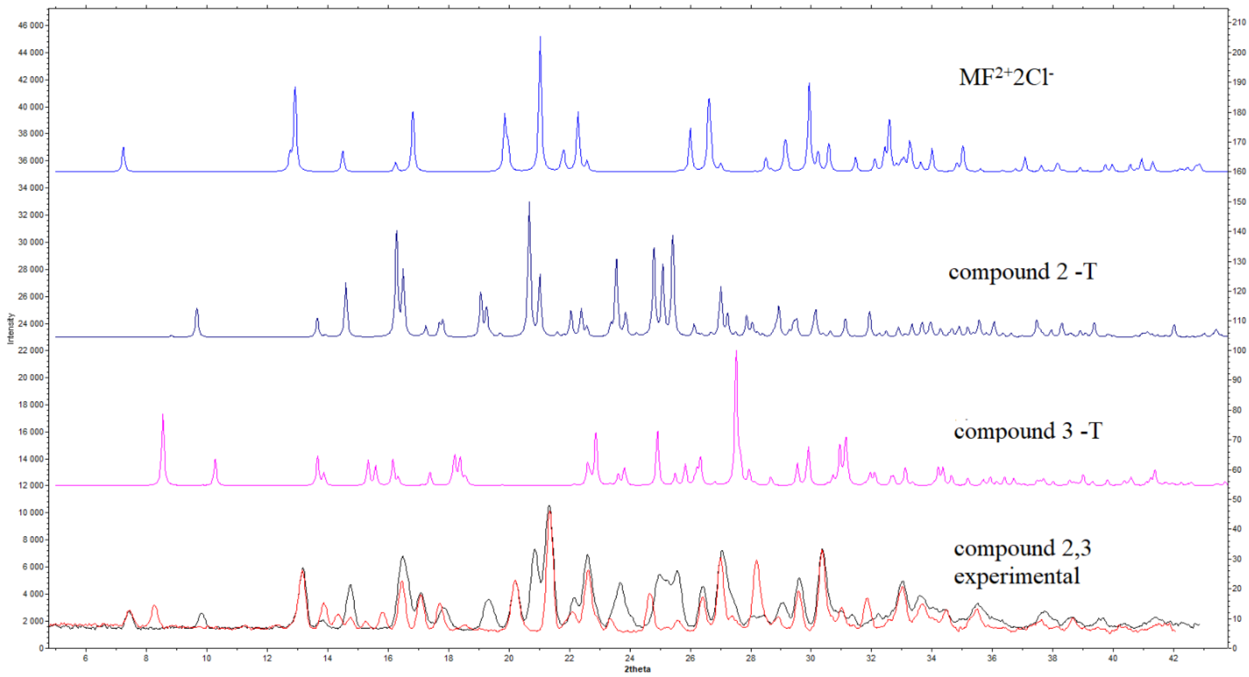

Figure S5. The PXR diffractogram of compounds **2** an **3** in different solvents (red- H_2O, black- DMSO) before the purification in comparison to theoretical ones: compound **2** -T **,** compound **3** -T **and** $\mathbf{MF^{2+}2Cl^-}$

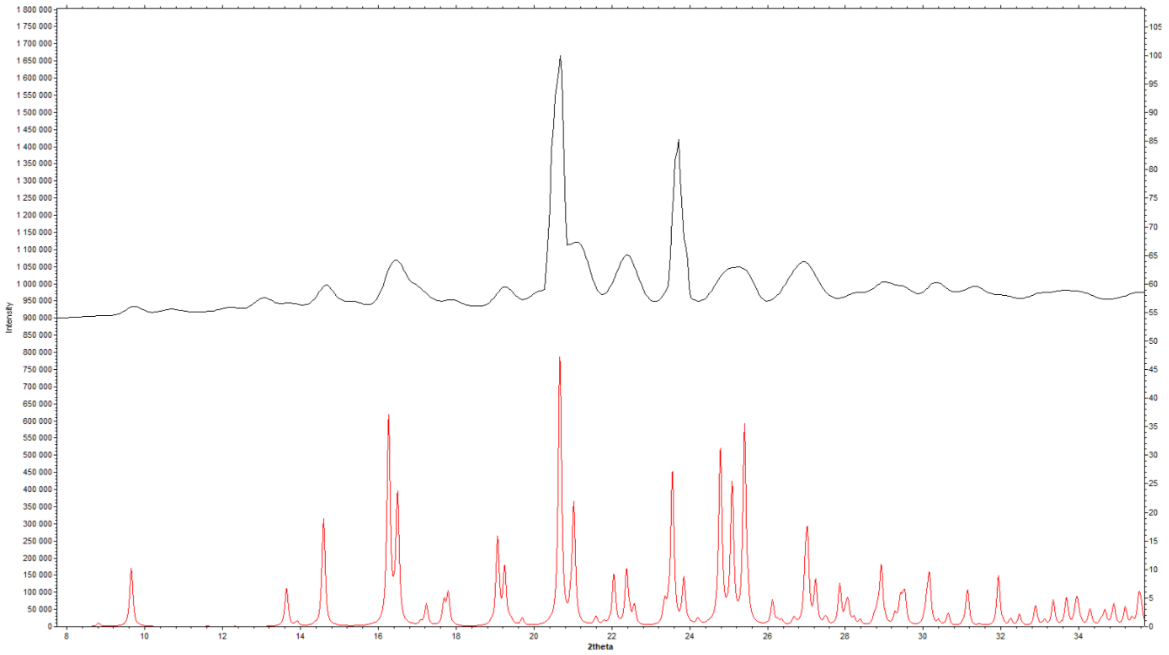

Figure S6. The PXR diffractogram of compound **2**; the results of the mechanochemistry reaction after the purification (black experimental , red theoretical one)

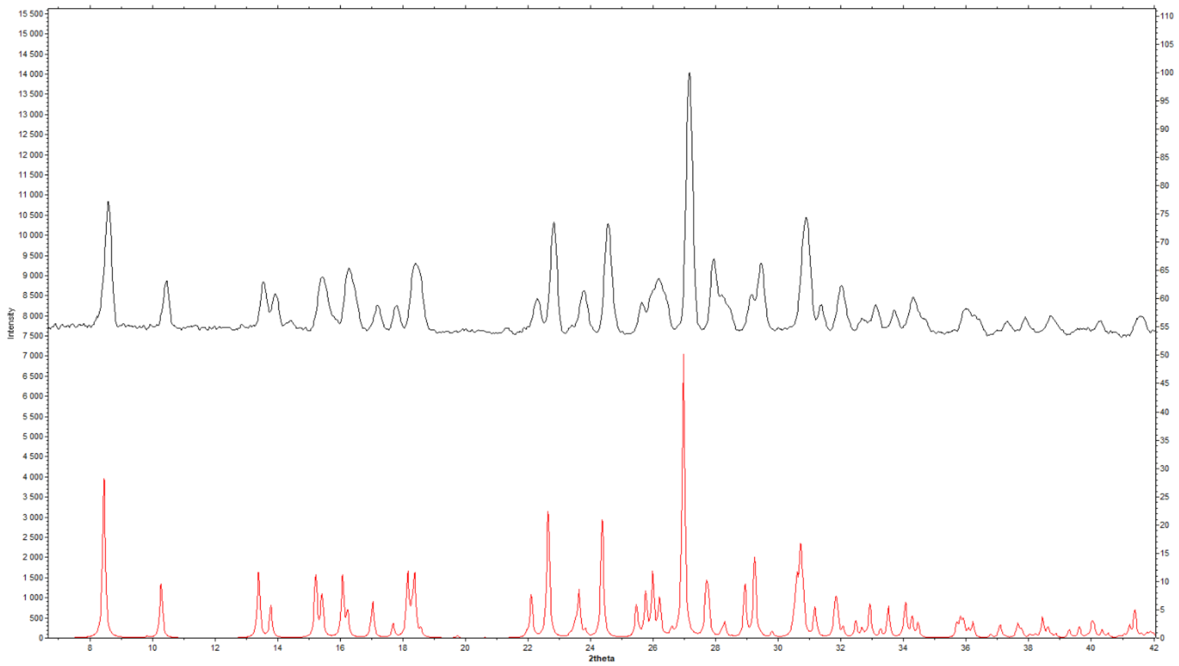

Figure S7. The PXR diffractogram of compound **3**; the results of the mechanochemistry reaction after the purification (black experimental , red theoretical one)

Table S4 Characteristics of bond critical points found for compound **1**

Atom1	Atom2	sym2	Gcp	Vcp	D12	D1	D2	DEN	LAPL	Λ_1	Λ_2	Λ_3	Eliip	type
O23	C22	55501	1309,42	-3291,67	1,2116	0,7909	0,4207	2,81732	-24,703	-27,63	-25,95	28,87	0,0646	(3,-1)
O13	C12	55501	1290,33	-3272,8	1,214	0,7917	0,4223	2,81134	-25,412	-27,43	-25,76	27,78	0,0647	(3,-1)
O33	C32	55501	1259,23	-3238,08	1,2176	0,793	0,4245	2,79901	-26,421	-27,19	-25,48	26,25	0,0673	(3,-1)
O25	C24	55501	1090,39	-3047,31	1,2401	0,7984	0,4417	2,72997	-31,815	-25,67	-23,78	17,63	0,0797	(3,-1)
O15	C14	55501	1040,13	-2985,46	1,2484	0,7981	0,4503	2,70586	-33,234	-25,08	-23,16	15,01	0,0832	(3,-1)
O16	C14	55501	1029,03	-2972,5	1,251	0,7976	0,4534	2,70098	-33,574	-24,84	-22,98	14,25	0,0808	(3,-1)
O26	C24	55501	979,58	-2894,38	1,2626	0,7946	0,4681	2,66562	-34,337	-24,13	-22,14	11,93	0,0898	(3,-1)
O31	C32	55501	954,69	-2789,23	1,2933	0,793	0,5004	2,60348	-32,304	-23,19	-20,49	11,37	0,1321	(3,-1)
O1w	H1w	55501	431,55	-2519,47	0,9583	0,7389	0,2194	2,59794	-60,814	-46,72	-46,21	32,12	0,011	(3,-1)
O11	C12	55501	953,03	-2763,54	1,298	0,7907	0,5073	2,58664	-31,482	-22,97	-20,23	11,72	0,1356	(3,-1)
O21	H21	55501	411,35	-2448,45	0,9674	0,7448	0,2227	2,5565	-59,689	-45,25	-45,12	30,68	0,0027	(3,-1)
O21	C22	55501	957,41	-2706,62	1,31	0,7856	0,5244	2,54632	-29,071	-22,4	-19,61	12,94	0,1421	(3,-1)
O11	H11	55501	453,29	-2374,76	0,9821	0,7484	0,2337	2,4912	-53,904	-42,32	-42,18	30,59	0,0034	(3,-1)
N2	C4	55501	916,31	-2603,48	1,3234	0,7992	0,5242	2,48926	-28,302	-20,45	-17,37	9,52	0,1777	(3,-1)
N9	H9b	55501	541,86	-2416,22	0,9674	0,7308	0,2366	2,48923	-48,922	-38,29	-36,58	25,95	0,0466	(3,-1)
O31	H31	55501	457,29	-2372.48	0,983	0,7486	0,2344	2,48822	-53,527	-42,16	-41,97	30,61	0,0045	(3,-1)
N5	H5b	55501	542,62	-2412,21	0,9681	0,731	0,2371	2,48618	-48,72	-37,75	-36,92	25,96	0,0225	(3,-1)
O1w	H2w	55501	491,65	-2377,78	0,984	0,746	0,2379	2,47994	-51,198	-41,44	-41,39	31,63	0,0013	(3,-1)
N5	C4	55501	912,9	-2569,69	1,3174	0,7893	0,5281	2,46685	-27,312	-20,2	-17,1	9,99	0,1816	(3,-1)
N8	C7	55501	910,36	-2560,59	1,3183	0,7886	0,5297	2,46137	-27,164	-20,16	-17,07	10,07	0,1813	(3,-1)
N9	C7	55501	910,13	-2555,86	1,3198	0,7888	0,531	2,45813	-27,007	-20,14	-17,01	10,14	0,1838	(3,-1)
N6	C7	55501	878,32	-2355,81	1,3683	0,7998	0,5687	2,32648	-21,998	-18,44	-15,88	12,32	0,1614	(3,-1)
N5	H5a	55501	556,65	-2193,64	1,0055	0,7457	0,2598	2,32551	-39,665	-33,25	-31,66	25,24	0,0501	(3,-1)

N9	H9a	55501	554,88	-2148,02	1,0141	0,7495	0,2646	2,29277	-38,119	-32,36	-30,79	25,03	0,0509	(3,-1)
N6	C4	55501	870,93	-2300,7	1,3801	0,7995	0,5809	2,28883	-20,517	-17,91	-15,68	13,08	0,1418	(3,-1)
N8	H8b	55501	555,65	-2138,6	1,0159	0,75	0,2659	2,28555	-37,717	-32,11	-30,58	24,98	0,05	(3,-1)
N6	H6	55501	572,52	-2093,42	1,0367	0,7552	0,2814	2,2458	-34,82	-30,35	-28,83	24,36	0,0529	(3,-1)
N8	H8a	55501	554,26	-2080,22	1,0261	0,7541	0,2721	2,24277	-35,676	-31	-29,44	24,76	0,0529	(3,-1)
C1	H1b	55501	602,62	-1782,33	1,0492	0,6695	0,3796	1,99302	-21,188	-18,82	-17,88	15,5	0,0526	(3,-1)
C3	H3c	55501	588,07	-1726,1	1,0612	0,6773	0,384	1,95322	-20,192	-18,36	-17,37	15,54	0,0568	(3,-1)
C1	H1c	55501	581,97	-1694	1,0699	0,6836	0,3864	1,92935	-19,461	-18,06	-17,03	15,62	0,0602	(3,-1)
C3	H3a	55501	570,42	-1648,13	1,0806	0,6905	0,3902	1,89608	-18,626	-17,42	-16,84	15,63	0,0344	(3,-1)
C1	H1a	55501	551,72	-1569,52	1,1013	0,7042	0,3972	1,83766	-17,112	-16,73	-16,03	15,66	0,0437	(3,-1)
C3	H3b	55501	547,78	-1555,76	1,1047	0,706	0,3987	1,8276	-16,896	-16,38	-16,13	15,61	0,0155	(3,-1)
C32	C32	75751	562,28	-1482,55	1,545	0,7725	0,7725	1,75788	-13,144	-13,13	-11,09	11,08	0,1843	(3,-1)
C14	C12	55501	558,22	-1472,37	1,5501	0,7753	0,7751	1,75071	-13,068	-13,05	-11,07	11,05	0,178	(3,-1)
N2	C1	55501	612,94	-1489,14	1,457	0,8497	0,6073	1,74138	-9,666	-12,02	-10,93	13,29	0,0995	(3,-1)
C22	C24	55501	553,28	-1455,86	1,5537	0,7768	0,7771	1,73833	-12,825	-12,98	-10,93	11,08	0,1882	(3,-1)
C24	C22	45501	553,28	-1455,86	1,5537	0,7771	0,7768	1,73833	-12,825	-12,98	-10,93	11,08	0,1882	(3,-1)
N2	C3	55501	608,51	-1477,58	1,4589	0,8502	0,6087	1,7331	-9,566	-11,95	-10,92	13,3	0,0943	(3,-1)
H31	O15	55501	120,95	-193,68	1,541	0,487	1,0547	0,46977	1,77	-3,74	-3,7	9,21	0,013	(3,-1)
H11	O16	45501	109,18	-175,73	1,5714	0,5027	1,0688	0,44376	1,565	-3,47	-3,46	8,5	0,0015	(3,-1)
O26	H21	65501	94,32	-148,76	1,6251	1,0916	0,5336	0,39931	1,464	-2,99	-2,98	7,43	0,0031	(3,-1)
O26	H6	55501	95,2	-124,52	1,6797	1,1096	0,571	0,33791	2,419	-2,27	-2,27	6,96	0,0005	(3,-1)
O25	H2w	55501	60,29	-85,08	1,819	1,1452	0,6739	0,27613	1,303	-1,78	-1,76	4,84	0,0102	(3,-1)
O1w	H8b	77651	59,73	-66,13	1,8563	1,2065	0,65	0,21522	1,958	-1,26	-1,25	4,47	0,0118	(3,-1)
H8b	O1w	77651	59,73	-66,13	1,8563	0,65	1,2065	0,21522	1,958	-1,26	-1,25	4,47	0,0118	(3,-1)
H5a	O16	55501	60,27	-65,9	1,8468	0,653	1,1949	0,21342	2,006	-1,25	-1,22	4,48	0,0281	(3,-1)
O25	H8a	67651	56,3	-58,63	1,8853	1,2143	0,672	0,19389	1,982	-1,11	-1,02	4,11	0,0909	(3,-1)
H5b	O33	66751	45,56	-48,08	1,9368	0,7151	1,2222	0,1734	1,58	-0,95	-0,93	3,45	0,021	(3,-1)
O13	H9a	45501	40,57	-41,59	1,9626	1,2312	0,732	0,15641	1,452	-0,84	-0,8	3,1	0,0431	(3,-1)
O31	H9b	54501	29,43	-26,06	2,1668	1,3372	0,832	0,10717	1,204	-0,49	-0,44	2,13	0,0941	(3,-1)

H1w	O33	56401	24,45	-22,95	2,2581	0,9329	1,3333	0,10358	0,953	-0,48	-0,46	1,9	0,0403	(3,-1)
O33	H9b	76751	21,28	-16,63	2,3298	1,4102	0,9256	0,07307	0,952	-0,29	-0,2	1,45	0,4736	(3,-1)
O31	O11	66501	24,08	-17,87	2,8764	1,5229	1,4001	0,07186	1,112	-0,23	-0,11	1,44	1,1576	(3,-1)
O15	O31	45501	21,83	-16,14	2,9587	1,4133	1,6621	0,06725	1,011	-0,18	-0,08	1,27	1,3511	(3,-1)
O13	H1c	55501	16	-12,38	2,4658	1,4491	1,0167	0,06054	0,721	-0,23	-0,23	1,18	0,0396	(3,-1)
O16	C1	45501	15,46	-10,59	3,1604	1,5001	1,6816	0,04689	0,747	-0,11	-0,07	0,93	0,7119	(3,-1)
O13	C12	56751	11,84	-8,76	3,2357	1,6367	1,6456	0,04668	0,548	-0,09	-0,03	0,67	1,504	(3,-1)
O23	N2	55501	14,28	-9,62	3,142	1,5048	1,6588	0,04301	0,695	-0,12	-0,06	0,87	sty.20	(3,-1)
O15	N9	66751	14,2	-9,33	3,0713	1,4986	1,5755	0,0404	0,7	-0,12	-0,07	0,88	0,7018	(3,-1)
O16	O11	56751	11,77	-7,75	3,1371	1,5512	1,6014	0,03628	0,58	-0,09	-0,05	0,72	0,8072	(3,-1)
C22	H1a	66651	8	-5,86	2,8293	1,7442	1,1554	0,03619	0,372	-0,1	-0,07	0,54	0,4904	(3,-1)
O1w	O31	76651	12,95	-8,29	3,1169	1,5848	1,5446	0,03572	0,647	-0,1	-0,06	0,81	0,6349	(3,-1)
O21	O21	57651	12,04	-7,77	3,0964	1,5482	1,5482	0,03491	0,599	-0,1	-0,08	0,78	0,1371	(3,-1)
O13	N5	45501	10,18	-6,65	3,2909	1,585	1,723	0,03253	0,504	-0,07	-0,05	0,62	0,2371	(3,-1)
H3a	O26	76651	8,97	-5,76	2,7699	1,1691	1,606	0,02892	0,447	-0,07	-0,07	0,59	0,1172	(3,-1)
H3b	H3b	76651	9,92	-6,08	2,6887	1,3444	1,3444	0,02665	0,506	-0,08	-0,02	0,6	2,4001	(3,-1)
N5	N9	45501	8,37	-5,29	3,3625	1,6883	1,6757	0,02651	0,421	-0,07	-0,05	0,53	0,518	(3,-1)
H3c	O23	65501	6	-4,02	2,8732	1,2449	1,6296	0,02527	0,293	-0,09	-0,08	0,46	0,025	(3,-1)
O1w	H1b	76651	6,55	-4,28	2,8507	1,631	1,225	0,02506	0,324	-0,09	-0,08	0,49	0,0792	(3,-1)
H3a	O21	66651	7,89	-4,88	2,8722	1,2108	1,6682	0,02389	0,4	-0,06	-0,03	0,5	0,7632	(3,-1)
O11	O1w	66651	6,93	-4,33	3,3994	1,7696	1,6697	0,02292	0,35	-0,05	-0,03	0,43	0,6362	(3,-1)
O16	O16	66751	6,83	-4,17	3,3133	1,6567	1,6567	0,02102	0,349	-0,05	-0,02	0,42	2,2508	(3,-1)
O11	N5	56751	7,3	-4,35	3,3668	1,6576	1,7325	0,02013	0,376	-0,05	-0,01	0,44	3,4473	(3,-1)

Symm: Ortep symmetry code; Gcp: kinetic energy density (kJ/mol/Bohr^3); Vcp: potential energy density(kJ/mol/Bohr^3); D12: distance between atoms 1 and 2 (Å); D1: distance between atom 1 and CP (Å); D2: distance between atom 2 and CP (Å), LAP: laplacian (eÅ^{-5})]; RHO: electron density (eÅ^{-3})]; λ1, λ2, λ3 :Hassian matrix, ELLIP – ellipticity

Table S5 Characteristics of bond critical points found for compound **2**

Atom1	Atom2	symm	Gcp	Vcp	D12	D1	D2	DEN	LAPL	Λ_1	Λ_2	Λ_3	Eliip	type
C16	O17	55501	1241,74	-3208,14	1,2234	0,4258	0,7979	2,78535	26,606	-26,99	-25,27	25,65	0,0679	(3,-1)
O27	C26	55501	1217,17	-3181,02	1,2266	0,799	0,4278	2,77575	27,414	-26,78	-25,04	24,41	0,0694	(3,-1)
O23	C22	55501	1167,52	-3131,61	1,2375	0,8046	0,4331	2,7598	29,246	-26,19	-24,44	21,38	0,0718	(3,-1)
O13	C12	55501	1117,28	-3074,38	1,2445	0,8058	0,4389	2,73888	30,834	-25,74	-23,89	18,8	0,0772	(3,-1)
O11	C12	55501	1027,5	-2963,33	1,2597	0,8058	0,454	2,69544	33,349	-24,71	-22,78	14,15	0,0848	(3,-1)
O21Aa	C22	55501	959,5	-2815,44	1,2754	0,7855	0,4902	2,61953	32,913	-23,15	-20,5	10,74	0,1293	(3,-1)
O18	C16	55501	943,27	-2780,53	1,2889	0,7944	0,4947	2,60146	32,823	-23,4	-20,64	11,22	0,1338	(3,-1)
O28	C26	55501	940,43	-2757,69	1,2929	0,7922	0,5008	2,58695	32,193	-23,21	-20,4	11,42	0,1376	(3,-1)
N2	C4	55501	908,39	-2614,27	1,315	0,8029	0,5121	2,49952	29,28	-20,67	-17,56	8,95	0,1775	(3,-1)
N8	C7	55501	899,3	-2543,73	1,3164	0,7892	0,5271	2,45341	27,358	-20,18	-17,06	9,88	0,1832	(3,-1)
N9	C7	55501	887,61	-2490,73	1,3187	0,7917	0,5271	2,42011	26,27	-19,63	-16,61	9,97	0,1814	(3,-1)
N5	C4	55501	885,57	-2473,13	1,3215	0,7905	0,5314	2,40833	25,774	-19,42	-16,57	10,21	0,1717	(3,-1)
N6	C7	55501	884,7	-2397,84	1,3644	0,798	0,5665	2,35466	23,073	-19,05	-16,11	12,09	0,1822	(3,-1)
N6	H6	55501	519,64	-2214,18	0,9959	0,7427	0,2532	2,35384	43,136	-34,55	-32,96	24,37	0,0482	(3,-1)
C25	C24	55501	820,08	-2272,7	1,3379	0,6697	0,6694	2,28694	23,224	-17,71	-14,71	9,19	0,2038	(3,-1)
N9	H9b	55501	499,21	-2103,08	1,007	0,7514	0,2556	2,28017	40,558	-33,2	-31,6	24,24	0,0506	(3,-1)
N6	C4	55501	867,26	-2279,46	1,3891	0,7976	0,5915	2,27452	20,007	-17,62	-15,98	13,59	0,1029	(3,-1)
C15	C14	55501	810,87	-2235,01	1,3459	0,6737	0,6734	2,26247	22,516	-17,46	-14,5	9,44	0,2036	(3,-1)
N8	H8b	55501	504,89	-2019,22	1,0233	0,7578	0,2656	2,21556	37,062	-31,25	-29,76	23,95	0,0498	(3,-1)
N5	H5b	55501	503,44	-2010,3	1,0237	0,7578	0,266	2,20938	36,841	-31,04	-29,77	23,96	0,0427	(3,-1)
N5	H5a	55501	503,67	-2004,29	1,0251	0,7583	0,2668	2,20477	36,603	-30,87	-29,62	23,89	0,0423	(3,-1)
N8	H8a	55501	505,21	-1977,27	1,029	0,7593	0,2698	2,18371	35,498	-30,45	-28,91	23,87	0,0534	(3,-1)

N9	H9a	55501	506,97	-1966,17	1,0313	0,7602	0,2713	2,1746	34,961	-30,17	-28,63	23,83	0,054	(3,-1)
C16	O17	55501	1266,15	-2314,74	1,2234	0,4316	0,7918	2,15088	7,988	-11,76	-11,49	31,24	0,0233	(3,-1)
O27	C26	55501	1242,54	-2289,02	1,2266	0,7932	0,4334	2,14019	7,198	-11,6	-11,41	30,21	0,0164	(3,-1)
O18	H11	55501	509,41	-1902,84	1,06	0,773	0,287	2,12505	32,456	-30,52	-30,27	28,33	0,0084	(3,-1)
O23	C22	55501	1166,41	-2206,28	1,2375	0,7975	0,44	2,10561	4,646	-11,27	-10,96	26,87	0,0281	(3,-1)
O13	C12	55501	1120,43	-2155,49	1,2444	0,7997	0,4447	2,08389	3,135	-11,04	-10,73	24,91	0,0291	(3,-1)
C3	H3a	55501	621,67	-1903,29	1,014	0,6511	0,363	2,08169	24,231	-20,31	-19,09	15,17	0,0643	(3,-1)
C1	H1c	55501	617,39	-1877,77	1,0208	0,6561	0,3648	2,06329	23,607	-20,03	-18,83	15,25	0,0638	(3,-1)
O11	C12	55501	1030,4	-2050,87	1,2598	0,804	0,4558	2,03682	0,365	-10,62	-10,31	21,29	0,0301	(3,-1)
C1	H1b	55501	602,7	-1824,4	1,0313	0,6628	0,3685	2,02676	22,727	-19,54	-18,52	15,33	0,0553	(3,-1)
C3	H3b	55501	605,18	-1825,54	1,0289	0,6613	0,3679	2,02668	22,587	-19,65	-18,31	15,38	0,0732	(3,-1)
C3	H3c	55501	596,55	-1775,89	1,0453	0,6737	0,3716	1,99026	21,397	-19	-17,94	15,55	0,0591	(3,-1)
O28	H28	55501	520,73	-1731,95	1,0957	0,7832	0,3125	1,98539	25,352	-26,48	-26,46	27,58	0,0007	(3,-1)
O21Aa	C22	55501	923,55	-1913,09	1,2751	0,7986	0,4771	1,96893	-2,423	-9,86	-9,67	17,1	0,0197	(3,-1)
O18	C16	55501	907,83	-1888,23	1,2888	0,8084	0,4804	1,95506	-2,664	-10,02	-9,75	17,11	0,0277	(3,-1)
C1	H1a	55501	578,95	-1712,61	1,0574	0,6808	0,3767	1,94591	20,366	-18,51	-17,39	15,54	0,0644	(3,-1)
O28	C26	55501	893,21	-1864,91	1,2929	0,8087	0,4842	1,94194	-2,881	-9,92	-9,75	16,79	0,0181	(3,-1)
C16	C15	55501	633,48	-1672,58	1,4835	0,7351	0,7487	1,89016	14,892	-14,17	-11,9	11,18	0,1912	(3,-1)
C26	C25	55501	632,98	-1671,83	1,4835	0,7353	0,7487	1,88974	14,901	-14,19	-11,88	11,17	0,194	(3,-1)
C24	C22	55501	628,95	-1654,54	1,4886	0,751	0,7384	1,87694	14,563	-14,03	-11,76	11,23	0,193	(3,-1)
C12	C14	55501	627,59	-1651,78	1,489	0,7389	0,7506	1,87519	14,561	-14,04	-11,76	11,23	0,1939	(3,-1)
C12	C14	56552	627,59	-1651,78	1,489	0,7389	0,7506	1,87519	14,561	-14,04	-11,76	11,23	0,1939	(3,-1)
C15	H15	55501	523,44	-1592,15	1,0796	0,709	0,3708	1,86882	20,019	-18,04	-17,09	15,12	0,0555	(3,-1)
N2	C4	55501	788,5	-1715,85	1,315	0,7745	0,5405	1,86138	-5,098	-9,47	-9,21	13,58	0,0288	(3,-1)
N8	C7	55501	788,32	-1712,71	1,3165	0,7745	0,542	1,85879	-4,996	-9,37	-9,3	13,67	0,0071	(3,-1)
N9	C7	55501	787,96	-1707,52	1,3187	0,7746	0,5441	1,85456	-4,832	-9,38	-9,21	13,76	0,0184	(3,-1)
N5	C4	55501	782,6	-1693,43	1,3215	0,7751	0,5464	1,84487	-4,708	-9,41	-9,12	13,82	0,0309	(3,-1)
C14	H14	55501	505,94	-1518,96	1,0991	0,7212	0,3779	1,81398	18,617	-17,31	-16,37	15,06	0,0574	(3,-1)
C25	H25	55501	505,86	-1516,56	1,0996	0,7217	0,378	1,81195	18,535	-17,27	-16,35	15,08	0,0567	(3,-1)

C24	H24	55501	503,73	-1508,52	1,1008	0,7223	0,3786	1,80594	18,396	-17,2	-16,28	15,08	0,0563	(3,-1)
N2	C3	55501	608,72	-1473,49	1,4594	0,849	0,6104	1,72938	-9,401	-11,94	-10,88	13,42	0,0978	(3,-1)
N2	C1	55501	605,48	-1463,91	1,4617	0,85	0,6117	1,72231	-9,287	-11,89	-10,83	13,43	0,098	(3,-1)
N6	C7	55501	734,47	-1524,08	1,3644	0,7796	0,5849	1,71846	-2,024	-8,76	-8,65	15,39	0,0132	(3,-1)
N6	C4	55501	706,06	-1430,38	1,3891	0,7843	0,6048	1,6466	-0,671	-8,46	-8,23	16,02	0,0285	(3,-1)
C25	C24	55501	613,83	-1358,96	1,3382	0,6692	0,669	1,62329	-4,821	-7,77	-7,64	10,58	0,0169	(3,-1)
C15	C14	55501	606,18	-1334,16	1,3459	0,6729	0,673	1,60378	-4,472	-7,67	-7,53	10,72	0,0183	(3,-1)
O18	H11	55501	532,5	-1257,92	1,018	0,7877	0,2303	1,56673	-7,083	-19,12	-18,98	31,02	0,0076	(3,-1)
O28	H28	55501	532,77	-1257,97	1,018	0,7876	0,2304	1,56665	-7,065	-19,09	-19,01	31,03	0,0041	(3,-1)
N8	H8a	55501	499,9	-1222,95	1,015	0,7661	0,2488	1,54896	-8,193	-16,86	-16,73	25,4	0,0073	(3,-1)
N6	H6	55501	499,3	-1222,6	1,015	0,7664	0,2486	1,54892	-8,224	-16,86	-16,73	25,36	0,0076	(3,-1)
N9	H9a	55501	499,99	-1222,75	1,015	0,7661	0,2489	1,54873	-8,179	-16,85	-16,73	25,4	0,0073	(3,-1)
N8	H8b	55501	499,43	-1221,69	1,015	0,766	0,249	1,54798	-8,181	-16,84	-16,74	25,4	0,0062	(3,-1)
N5	H5b	55501	497,95	-1220,53	1,015	0,7664	0,2486	1,54758	-8,247	-16,87	-16,75	25,37	0,0071	(3,-1)
N5	H5a	55501	497,29	-1218,6	1,015	0,7661	0,2489	1,54605	-8,225	-16,84	-16,75	25,36	0,0054	(3,-1)
N9	H9b	55501	496,91	-1218,34	1,015	0,7663	0,2488	1,54599	-8,243	-16,84	-16,77	25,36	0,0045	(3,-1)
N2	C3	55501	626,54	-1192,36	1,4592	0,8025	0,6567	1,45745	2,229	-7,33	-7,28	16,83	0,0067	(3,-1)
N2	C1	55501	623,98	-1184,53	1,462	0,8034	0,6586	1,45093	2,328	-7,32	-7,19	16,84	0,0173	(3,-1)
C16	C15	55501	475,69	-943,7	1,4835	0,7416	0,7419	1,27763	0,282	-5,86	-5,79	11,93	0,0132	(3,-1)
C26	C25	55501	475,7	-943,66	1,4835	0,7416	0,7419	1,27757	0,284	-5,91	-5,74	11,93	0,0302	(3,-1)
C24	C22	55501	471,59	-932,04	1,4884	0,7444	0,744	1,26716	0,409	-5,82	-5,71	11,94	0,0191	(3,-1)
C12	C14	55501	470,31	-929,51	1,4889	0,7444	0,7445	1,26509	0,408	-5,86	-5,67	11,93	0,034	(3,-1)
C25	H25	55501	364,56	-850,42	1,083	0,7412	0,3418	1,23622	-4,454	-9,43	-9,42	14,39	0,0004	(3,-1)
C14	H14	55501	364,6	-850,42	1,083	0,7411	0,342	1,2362	-4,451	-9,49	-9,35	14,39	0,0143	(3,-1)
C15	H15	55501	364,42	-850,14	1,083	0,7411	0,3419	1,23598	-4,454	-9,5	-9,35	14,39	0,0162	(3,-1)
C24	H24	55501	364,38	-850,08	1,083	0,7411	0,3419	1,23594	-4,455	-9,45	-9,39	14,39	0,0068	(3,-1)
C3	H3c	55501	359,06	-827,34	1,095	0,7487	0,3463	1,2135	-4,01	-9,14	-9,13	14,26	0,0006	(3,-1)
C1	H1a	55501	358,62	-826,37	1,095	0,7484	0,3466	1,21267	-4,006	-9,13	-9,12	14,25	0,0012	(3,-1)
C1	H1c	55501	358,34	-826,12	1,095	0,7486	0,3464	1,21254	-4,018	-9,15	-9,13	14,25	0,0022	(3,-1)

C3	H3a	55501	357,86	-825,08	1,095	0,7484	0,3466	1,21164	-4,015	-9,13	-9,13	14,24	0,0004	(3,-1)
C3	H3b	55501	357,48	-824,47	1,095	0,7484	0,3466	1,21117	-4,021	-9,14	-9,12	14,24	0,002	(3,-1)
C1	H1b	55501	357,32	-824,11	1,095	0,7483	0,3467	1,21086	-4,019	-9,14	-9,13	14,24	0,0012	(3,-1)
O21Aa	H28	55501	195,55	-328,78	1,3658	0,9843	0,3817	0,65365	2,288	-6,13	-6,04	14,46	0,0154	(3,-1)
H28	O21Aa	55501	225,28	-330,02	1,4442	0,4874	0,9576	0,63024	4,426	-4,02	-3,95	12,4	0,0182	(3,-1)
O11	H11	55501	222,82	-326,44	1,45	0,9623	0,4878	0,62614	4,377	-4,11	-4,01	12,49	0,025	(3,-1)
H11	O11	56552	175,59	-294,48	1,4082	0,3998	1,0085	0,61146	2,081	-5,58	-5,58	13,23	0,0004	(3,-1)
O11	H11	46452	175,59	-294,48	1,4082	1,0085	0,3998	0,61146	2,081	-5,58	-5,57	13,23	0,0031	(3,-1)
H9a	O23	55501	97,26	-113,32	1,7833	0,6835	1,0999	0,30455	2,982	-1,51	-1,5	5,99	0,01	(3,-1)
H8b	O13	55501	95,12	-110,28	1,7898	0,6874	1,1024	0,29895	2,936	-1,49	-1,47	5,89	0,0133	(3,-1)
O21Aa	H8a	55501	85,35	-96,25	1,8391	1,1256	0,7145	0,27199	2,733	-1,25	-1,24	5,22	0,0051	(3,-1)
H9a	O23	55501	74,2	-83,66	1,7677	0,5957	1,1724	0,25004	2,377	-1,57	-1,49	5,44	0,0588	(3,-1)
O11	H6	46452	61,23	-75,04	1,8375	1,1876	0,6502	0,2431	1,741	-1,48	-1,47	4,69	0,0128	(3,-1)
O13	H8b	55501	70,48	-79,56	1,7815	1,1762	0,6053	0,24272	2,255	-1,49	-1,46	5,21	0,0194	(3,-1)
O27	H5b	54602	68,28	-78,44	1,7826	1,1788	0,6043	0,24266	2,134	-1,53	-1,51	5,17	0,0137	(3,-1)
O13	H9b	46452	60,67	-68,5	1,8321	1,2005	0,6318	0,22193	1,94	-1,33	-1,3	4,57	0,0198	(3,-1)
O21Aa	H8a	55501	59,38	-67,32	1,8257	1,1954	0,6304	0,22003	1,889	-1,31	-1,26	4,46	0,0418	(3,-1)
H5a	O17	55502	43,69	-45,93	1,9459	0,6948	1,252	0,16835	1,522	-0,95	-0,93	3,4	0,0248	(3,-1)
H25	O18	55601	22,44	-17,21	2,3389	0,941	1,3993	0,07306	1,016	-0,29	-0,27	1,57	0,0822	(3,-1)
O11	H24	55401	17,45	-13,46	2,4172	1,4373	0,9813	0,06344	0,787	-0,24	-0,23	1,26	0,0479	(3,-1)
H1b	O27	66651	16,77	-12,63	2,4512	1,0086	1,4449	0,05938	0,768	-0,23	-0,22	1,21	0,0272	(3,-1)
O21Aa	H14	55501	14	-10,57	2,6955	1,5275	1,1699	0,05355	0,64	-0,15	-0,09	0,88	0,7834	(3,-1)
O28	H15	55501	14,29	-10,7	2,6246	1,4571	1,1703	0,05341	0,657	-0,16	-0,15	0,96	0,088	(3,-1)
N8	C3	55501	17,52	-11,99	3,1309	1,5568	1,5879	0,0504	0,847	-0,1	-0,06	1,01	0,6046	(3,-1)
O18	C26	56651	12,7	-8,88	3,0667	1,5093	1,5975	0,04356	0,607	-0,11	-0,04	0,76	1,5792	(3,-1)
H15	H3c	66651	8,17	-6,34	2,2161	1,104	1,1198	0,04066	0,367	-0,16	-0,16	0,68	0,0314	(3,-1)
O21Aa	H14	55501	10,01	-7,23	2,6785	1,5798	1,1002	0,04033	0,469	-0,14	-0,13	0,74	0,1068	(3,-1)
O28	H15	55501	11,27	-7,59	2,6274	1,5308	1,0998	0,03728	0,549	-0,14	-0,11	0,79	0,2693	(3,-1)
O11	O27	56651	11,64	-7,54	3,087	1,5515	1,5384	0,03455	0,578	-0,1	-0,07	0,75	0,3745	(3,-1)

H3b	O27	46552	12,69	-8,03	2,6809	1,1452	1,5463	0,0341	0,637	-0,11	-0,07	0,82	0,7313	(3,-1)
N9	H1a	56552	7,93	-5,57	2,8343	1,674	1,1628	0,03319	0,378	-0,11	-0,09	0,58	0,1381	(3,-1)
H3b	N9	56452	9,67	-6,36	2,8359	1,1613	1,678	0,03213	0,477	-0,1	-0,06	0,63	0,5741	(3,-1)
O17	H3a	66651	9,69	-5,94	2,8441	1,5965	1,3259	0,02629	0,494	-0,08	-0,04	0,62	1,109	(3,-1)
N2	O23	56452	7,49	-4,76	3,4195	1,7906	1,6399	0,02515	0,375	-0,06	-0,04	0,47	0,6041	(3,-1)
N5	C24	56452	6,01	-3,89	3,5243	1,7507	1,7819	0,0232	0,298	-0,04	-0,03	0,37	0,2554	(3,-1)
O28	H3c	66651	6,25	-3,92	2,8965	1,6659	1,2558	0,02177	0,315	-0,07	-0,02	0,4	2,1992	(3,-1)
C15	C15	56651	4,55	-3,02	3,7249	1,8624	1,8624	0,02088	0,224	-0,04	-0,01	0,27	3,1519	(3,-1)
N5	N9	46452	6,35	-3,89	3,4615	1,7543	1,7119	0,02049	0,323	-0,04	-0,04	0,4	0,1137	(3,-1)

Symm: Ortep symmetry code; Gcp: kinetic energy density (kJ/mol/Bohr^3); Vcp: potential energy density(kJ/mol/Bohr^3); D12: distance between atoms 1 and 2 (Å); D1: distance between atom 1 and CP (Å); D2: distance between atom 2 and CP (Å), LAP: laplacian (eÅ^{-5})]; RHO: electron density (eÅ^{-3})]; λ1, λ2, λ3 :Hassian matrix, ELLIP – ellipticity

Table S6 Characteristics of bond critical points found for compound **3**

Atom1	Atom2	sym2	Gcp	Vcp	D12	D1	D2	DEN	LAPL	Λ_1	Λ_2	Λ_3	Eliip	type
O17	C16	55501	1160,45	-3125,76	1,235	0,8016	0,4336	2,7583	-29,55	-26,26	-24,45	21,17	0,074	(3,-1)
O13	C12	55501	1114,91	-3078,64	1,2459	0,8063	0,4397	2,74243	-31,164	-25,7	-23,84	18,37	0,0779	(3,-1)
O11	C12	55501	972,06	-2873,62	1,2753	0,8015	0,4739	2,6543	-34,126	-23,79	-21,73	11,39	0,095	(3,-1)
O18	C16	55501	945,99	-2769,13	1,2933	0,7921	0,5014	2,59282	-32,205	-23,23	-20,42	11,44	0,1376	(3,-1)
O1w	H2W	55501	422,7	-2458,93	0,9631	0,7416	0,2216	2,55978	-59,241	-45,42	-45,37	31,55	0,0011	(3,-1)
N9	C7	55501	909,72	-2579,69	1,3127	0,7906	0,5221	2,47496	-27,912	-20,3	-17,26	9,65	0,1762	(3,-1)
N2	C4	55501	910,27	-2574,57	1,3262	0,7974	0,5289	2,47119	-27,684	-20,3	-17,17	9,79	0,1826	(3,-1)
O1w	H1W	55501	476,34	-2327,76	0,9872	0,7483	0,2389	2,45025	-50,486	-40,85	-40,73	31,09	0,0029	(3,-1)
N8	C7	55501	905,52	-2539,64	1,3212	0,788	0,5332	2,44834	-26,75	-20,09	-16,91	10,25	0,1881	(3,-1)

N5	C4	55501	904,41	-2533,78	1,3225	0,7881	0,5344	2,44461	-26,617	-20,03	-16,91	10,32	0,1846	(3,-1)
N5	H5B	55501	530,77	-2238,81	0,9924	0,7427	0,2497	2,36759	-43,224	-34,98	-33,36	25,12	0,0484	(3,-1)
N5	H5A	55501	534,08	-2199,66	0,999	0,745	0,254	2,33809	-41,543	-34,08	-32,47	25,01	0,0497	(3,-1)
N8	H8b	55501	533,48	-2188,21	1,0014	0,7463	0,2552	2,32999	-41,166	-33,8	-32,31	24,94	0,0462	(3,-1)
N6	C7	55501	872,77	-2339,41	1,3699	0,8001	0,5701	2,31654	-21,804	-18,35	-15,83	12,37	0,1594	(3,-1)
N6	C4	55501	872,3	-2328	1,3725	0,7997	0,5731	2,30837	-21,42	-18,26	-15,74	12,57	0,1604	(3,-1)
N9	H9A	55501	535,81	-2150,03	1,0085	0,7489	0,2596	2,30124	-39,594	-32,99	-31,41	24,81	0,0505	(3,-1)
C14	C15	55501	824,23	-2271,44	1,3423	0,6713	0,672	2,28448	-22,872	-17,61	-14,65	9,39	0,2023	(3,-1)
N8	H8A	55501	535,45	-2096,97	1,0174	0,7523	0,2651	2,26223	-37,672	-31,92	-30,38	24,62	0,0508	(3,-1)
N9	H9b	55501	537,88	-2005,65	1,0342	0,7585	0,2757	2,19286	-34,141	-29,96	-28,5	24,33	0,0513	(3,-1)
N6	H1	55501	554,01	-2010,57	1,0448	0,7592	0,2857	2,19047	-33,137	-29,31	-27,77	23,94	0,0555	(3,-1)
C1	H1A	55501	606,09	-1808,34	1,0409	0,666	0,3749	2,01255	-21,888	-19,14	-18,18	15,43	0,0533	(3,-1)
O18	H11	55501	547,82	-1745,85	1,1009	0,7824	0,3186	1,98568	-23,873	-26,09	-25,88	28,09	0,0081	(3,-1)
C15	H15	55501	551,53	-1683,58	1,0637	0,6955	0,3683	1,93331	-21,314	-18,74	-17,79	15,22	0,0534	(3,-1)
C3	H3A	55501	576,88	-1688,93	1,0679	0,6838	0,3842	1,92726	-19,649	-17,86	-17,34	15,54	0,0298	(3,-1)
C1	H1B	55501	574,38	-1672,56	1,0723	0,687	0,3854	1,91476	-19,231	-17,93	-16,89	15,59	0,0611	(3,-1)
C3	H3B	55501	572,34	-1669,85	1,0718	0,6861	0,3858	1,91335	-19,282	-17,91	-16,9	15,54	0,0598	(3,-1)
C16	C15	55501	635,06	-1670,93	1,487	0,7367	0,7507	1,88812	-14,716	-14,13	-11,85	11,26	0,1923	(3,-1)
C1	H1C	55501	564,01	-1633,76	1,0816	0,693	0,3887	1,88674	-18,568	-17,57	-16,6	15,6	0,0582	(3,-1)
C14	H14	55501	532,11	-1600,62	1,0844	0,709	0,3755	1,87231	-19,694	-17,94	-16,98	15,23	0,057	(3,-1)
C14	C12	55501	626,06	-1639,24	1,4951	0,7539	0,7418	1,86527	-14,213	-13,91	-11,65	11,34	0,1938	(3,-1)
C3	H3C	55501	555,94	-1601,33	1,0895	0,6979	0,3917	1,86286	-17,97	-17,24	-16,31	15,57	0,0569	(3,-1)
N2	C3	55501	604,26	-1469,57	1,4594	0,8509	0,6085	1,72789	-9,585	-11,96	-10,88	13,25	0,0991	(3,-1)
N2	C1	55501	605,76	-1470,14	1,4599	0,8503	0,6096	1,72773	-9,495	-11,95	-10,88	13,33	0,098	(3,-1)
O11	H11	55501	226,44	-384,2	1,339	0,9724	0,3667	0,71935	2,522	-6,98	-6,97	16,47	0,0012	(3,-1)
O1w	H1	55501	106,37	-143,21	1,6427	1,1016	0,5412	0,37137	2,552	-2,61	-2,61	7,77	0,001	(3,-1)
O13	H9b	55501	81,97	-96,15	1,737	1,1557	0,5814	0,2768	2,489	-1,75	-1,75	5,99	0,0011	(3,-1)
Cl1	H1W	55501	44,93	-66,33	2,1093	1,3736	0,7358	0,24126	0,864	-1,26	-1,26	3,39	0,0007	(3,-1)
Cl1	H2W	66551	41,28	-56,75	2,1637	1,3995	0,7666	0,21464	0,948	-1,07	-1,07	3,08	0,0025	(3,-1)

O17	H9A	56501	60,67	-62,57	1,87	1,2099	0,661	0,20053	2,158	-1,13	-1,06	4,35	0,0698	(3,-1)
H5A	O13	75651	48,91	-50,88	1,9166	0,6947	1,2237	0,17797	1,724	-0,96	-0,95	3,64	0,0055	(3,-1)
O11	H8b	55501	39,38	-40,81	2,003	1,273	0,7301	0,15556	1,394	-0,84	-0,83	3,06	0,0086	(3,-1)
O17	H5B	76651	36,38	-34,54	2,0479	1,2854	0,7656	0,13344	1,403	-0,68	-0,63	2,71	0,0682	(3,-1)
H8A	Cl1	66551	27,12	-28,78	2,3156	0,7988	1,5186	0,12782	0,935	-0,55	-0,54	2,03	0,0255	(3,-1)
Cl1	H3A	65551	18,85	-16,27	2,5869	1,626	0,962	0,07916	0,787	-0,29	-0,28	1,36	0,0115	(3,-1)
N9	N5	55501	26,55	-18,54	2,9313	1,4693	1,4722	0,06763	1,269	-0,16	-0,1	1,53	0,6616	(3,-1)
H15	Cl1	66651	14,2	-11,93	2,7034	1,0154	1,6883	0,06417	0,605	-0,22	-0,21	1,04	0,0206	(3,-1)
O1w	H14	55401	19,65	-14,48	2,4198	1,4378	0,9822	0,0628	0,911	-0,24	-0,21	1,37	0,1402	(3,-1)
Cl1	N6	45501	13,81	-10,1	3,3257	1,734	1,5929	0,05005	0,644	-0,12	-0,11	0,87	0,0914	(3,-1)
O13	H1A	65651	12,36	-8,61	2,6072	1,5237	1,0882	0,04249	0,592	-0,15	-0,12	0,87	0,299	(3,-1)
N2	O18	54501	13,85	-9,33	3,1446	1,6417	1,5099	0,04223	0,674	-0,11	-0,07	0,86	0,5585	(3,-1)
N8	C15	66651	10,52	-7,55	3,3091	1,6365	1,7434	0,04108	0,495	-0,1	-0,04	0,63	1,4167	(3,-1)
N5	O18	54501	12,41	-8,19	3,2035	1,6642	1,5471	0,03764	0,61	-0,1	-0,04	0,76	1,3681	(3,-1)
C12	O11	76651	9,69	-6,81	3,2739	1,6768	1,633	0,03743	0,462	-0,07	-0,03	0,56	1,5641	(3,-1)
O11	H3B	56501	8,77	-6,21	2,7295	1,5794	1,1539	0,03581	0,416	-0,12	-0,09	0,63	0,383	(3,-1)
O17	N9	76651	9,55	-6,32	3,2746	1,6334	1,6571	0,03244	0,469	-0,08	-0,03	0,58	1,206	(3,-1)
N9	O18	66651	9,21	-6,08	3,3174	1,714	1,6824	0,0315	0,453	-0,08	-0,02	0,55	2,6484	(3,-1)
O17	H1C	66651	7,63	-5,24	2,7527	1,5768	1,1806	0,03085	0,368	-0,11	-0,1	0,57	0,0559	(3,-1)
O11	O11	66651	9,4	-6,01	3,207	1,6035	1,6035	0,02935	0,47	-0,07	-0,06	0,61	0,1859	(3,-1)
H14	C3	75651	9,95	-6,23	2,931	1,1695	1,7758	0,02851	0,502	-0,09	-0,02	0,62	3,3722	(3,-1)
H1B	H3C	75551	6,42	-4,23	2,4129	1,2084	1,2108	0,02524	0,316	-0,1	-0,06	0,47	0,6219	(3,-1)
O13	O18	76651	7,25	-4,56	3,358	1,6562	1,7179	0,02401	0,365	-0,04	-0,04	0,44	0,1145	(3,-1)
N8	C15	76651	6,41	-4,13	3,5444	1,763	1,7905	0,02388	0,319	-0,05	-0,03	0,4	0,5454	(3,-1)
H3C	O1w	75551	8,34	-5,03	2,9148	1,3372	1,6346	0,02283	0,428	-0,06	-0,04	0,53	0,6278	(3,-1)
N2	C3	75551	6,33	-3,87	3,6201	1,775	1,8498	0,02018	0,323	-0,04	-0,02	0,39	0,9643	(3,-1)

Symm: Ortep symmetry code; Gcp: kinetic energy density (kJ/mol/Bohr^3); Vcp: potential energy density(kJ/mol/Bohr^3); D12: distance between atoms 1 and 2 (Å); D1: distance between atom 1 and CP (Å); D2: distance between atom 2 and CP (Å), LAP: laplacian (eÅ^{-5})]; RHO: electron density (eÅ^{-3})]; λ1, λ2, λ3 :Hassian matrix, ELLIP – ellipticity

Table S7. MIC values of gram-positive strains according to tested compounds.

Strains	MIC* of tested compounds (mg/L)		
	1	MF	OXALIC
Staphylococcus aureus ATCC 29213	>32	>32	>32
Staphylococcus aureus MRSA (clinical strain)	>32	>32	>32
Enterococcus faecium ATCC 700 221 (VRE)	>32	>32	>32
Enterococcus faecalis ATCC 19433	>32	>32	>32

**MIC – minimal inhibitory concentration*

Table S8. MIC values of gram-negative strains according to tested compounds

Strains	MIC of tested compounds (mg/L)		
	**1**	MF	OXALIC
Escherichia coli ATCC 25922	>32	>32	>32
Pseudomonas aeruginosa ATCC 27583	>32	>32	>32

**MIC – minimal inhibitory concentration*
